# SiMRiv: an R package for mechanistic simulation of individual, spatially-explicit multistate movements in rivers, heterogeneous and homogeneous spaces incorporating landscape bias

**DOI:** 10.1186/s40462-019-0154-8

**Published:** 2019-04-02

**Authors:** Lorenzo Quaglietta, Miguel Porto

**Affiliations:** 1 CIBIO/InBio, Centro de Investigação em Biodiversidade e Recursos Genéticos, Laboratório Associado, Universidade do Porto, Campus Agrário de Vairão, 4485–661 Vairão, Portugal; 2 CIBIO/InBio, Centro de Investigação em Biodiversidade e Recursos Genéticos, Laboratório Associado, Instituto Superior de Agronomia, Universidade de Lisboa, Tapada da Ajuda, 1349-017 Lisbon, Portugal

**Keywords:** Connectivity, Dendritic ecological networks (DENs), Landscape heterogeneity, Linear habitats, Hidden Markov models, Mechanistic movement models, Individual-based movement simulation, Movement ecology, River networks, Resistance

## Abstract

**Background:**

Lack of suitable analytical software and computational power constrains the comprehension of animal movement. In particular, we are aware of no tools allowing simulating spatially-explicit multistate Markovian movements constrained to linear features or conditioned by landscape heterogeneity, which hinders movement ecology research in linear/dendritic (e.g. river networks) and heterogeneous landscapes.

SiMRiv is a novel, fast and intuitive R package we designed to fill such gap. It does so by allowing continuous-space mechanistic spatially-explicit simulation of multistate Markovian individual movements incorporating landscape bias on local behavior.

**Results:**

We present SiMRiv and its main functionalities, illustrate its simulation capabilities and easy-of-use, and discuss its limitations and potential improvements. We further provide examples of use and a preliminary evaluation, using real and simulated data, of a parameter approximation experimental method. SiMRiv allowed us to generate increasingly complex movements of three theoretical species (aquatic, semiaquatic and terrestrial), showing the effects of input parameters and water-dependence on emerging movement patterns, and to parameterize a high-frequency simulation model from real, low-frequency movement (telemetry) data. Typical running times for conducting 1000 simulations with 10,000 steps each, of two-state movement trajectories in a river network, were of ca. 3 min in an Intel Core i7 CPU X990 @ 3.47 GHz.

**Conclusions:**

SiMRiv allows simulation of movements constrained to linear habitats or conditioned by landscape heterogeneity, therefore enhancing the application of movement ecology to linear/dendritic and heterogeneous landscapes. Importantly, the software is flexible enough to be used in linear, heterogeneous, as well as homogeneous landscapes. Using the same software, algorithm and approach, one can therefore use SiMRiv to study the movement of different organisms in a variety of landscapes, facilitating comparative research.

SiMRiv balances ease and speed with high realism of the movement models obtainable, constituting a fast, powerful, yet intuitive tool, which should contribute exploring several movement-related questions. Its applications depart from the generation of mechanistic null movement models, up to population level (e.g. landscape connectivity) analyses, holding potential for all fields requiring the simulation of random trajectories.

**Electronic supplementary material:**

The online version of this article (10.1186/s40462-019-0154-8) contains supplementary material, which is available to authorized users.

## Background

Scientists in a variety of fields often rely on simulations of random trajectories to make inference on the movement of organisms or cells e.g. [1–7]. In the burgeoning field of Movement Ecology, for instance, the generation of random trajectories is often the base for developing home range/movement models e.g. [3, 5], and assessing connectivity [7–9], site fidelity [6] and habitat selection [10]. Despite the availability of various ecological software simulating movements [11–16], we are aware of no available tools allowing spatially-explicit simulation of Markovian multistate correlated random walk movements constrained to linear features (e.g. rivers and other dendritic ecological networks – DENs [17] – as well as roads) or incorporating landscape bias. Thus, movements simulated using available software are not properly comparable with real movements of organisms moving in linear or heterogeneous landscapes, and therefore not suitable to be used as null models, hindering the testing of hypotheses on the mechanisms underlying movement behavior in non-homogeneous landscapes.

The highlighted lack of software might help explaining why individual-based movement models commonly used in ecological research so far have rarely been applied to freshwater DENs, compared to the number of studies focused on marine and 2-dimensional landscapes e.g. [18–21], possibly due to the analytical challenges imposed by DENs’ spatial configuration cf. [22–24], and focused more on homogeneous, featureless [25, 26] or largely homogeneous [8] landscapes. Similarly, while robust testing of site fidelity has been performed for organisms living in homogeneous, terrestrial landscapes e.g. [6, 27], similar studies in freshwaters have been simpler in their nature e.g. [28]. Thus, the deep comprehension of animal movement in linear (e.g. river networks) and secondarily heterogeneous landscapes remains limited.

SiMRiv is a novel free, open-source and user-friendly software (R package) for continuous-space mechanistic simulation of spatially-explicit, multistate (Markovian) individual movements incorporating landscape influence on local behavior, which we conceived to fill the highlighted software gap. SiMRiv so far allows simulating simple or composite random walk (RW) and correlated random walk (CRW) movements [29, 30] locally biased by landscape heterogeneity or constrained to specific landscape features (e.g. rivers). Hence, it allows simulation of movements of any organism in any landscape, including river networks. SiMRiv’s approach focuses on parameters with which researchers are already familiar, like landscape resistance layers, step length and turning angle (i.e. the distance and the angle between two successive locations, respectively). The influence of landscape resistance on animal movement and connectivity is a well-known and consolidated field of research e.g. [31, 32], as is the use of the step length and the turning angle in Movement Ecology e.g. [10, 29, 33, 34].

SiMRiv may be used as a process-based, mechanistic [3, 35, 36] movement simulation tool enabling simulation-based null model [37] hypothesis testing, as well as for population level analyses e.g. [38]. Here, we summarize the main novelties and functionalities of SiMRiv, illustrate software capability to simulate complex animal movements in linear, homogeneous and heterogeneous landscapes, show the effects of varying input parameters and animal behaviors on emerging movement patterns, and provide examples of use of the software. In addition, we provide a preliminary evaluation, using both real and simulated data, of an experimental method for parameterizing the simulation model from real (observed) data.

## Implementation

### SiMRiv’s algorithm description

One of SiMRiv’s primary features, and the main reason for which it was originally designed, is the ability to simulate movements in linear/dendritic (e.g. river networks) or heterogeneous landscapes - i.e. landscapes where the organism has varying degrees of permeability/affinity to distinct landscape features. Such spatially explicit permeability/affinity is emulated by the concept of “landscape resistance”, reflecting the willingness of an organism to cross a specific environment and the physiological cost of moving through it [32]. The pixel values of the user-provided resistance raster dictate how much the animal is attracted to or repelled by that pixel, and how difficult it is to move within it. At the most extreme case, the resistance raster may consist of only 0 s (no resistance) and 1 s (infinite resistance), which is adequate, for example, to simulate a fish in a river. At the other extreme, simulations may be conducted without any raster, i.e. in a homogeneous space, as in previously available software. All intermediate situations are nevertheless possible.

**Fig. 1:**
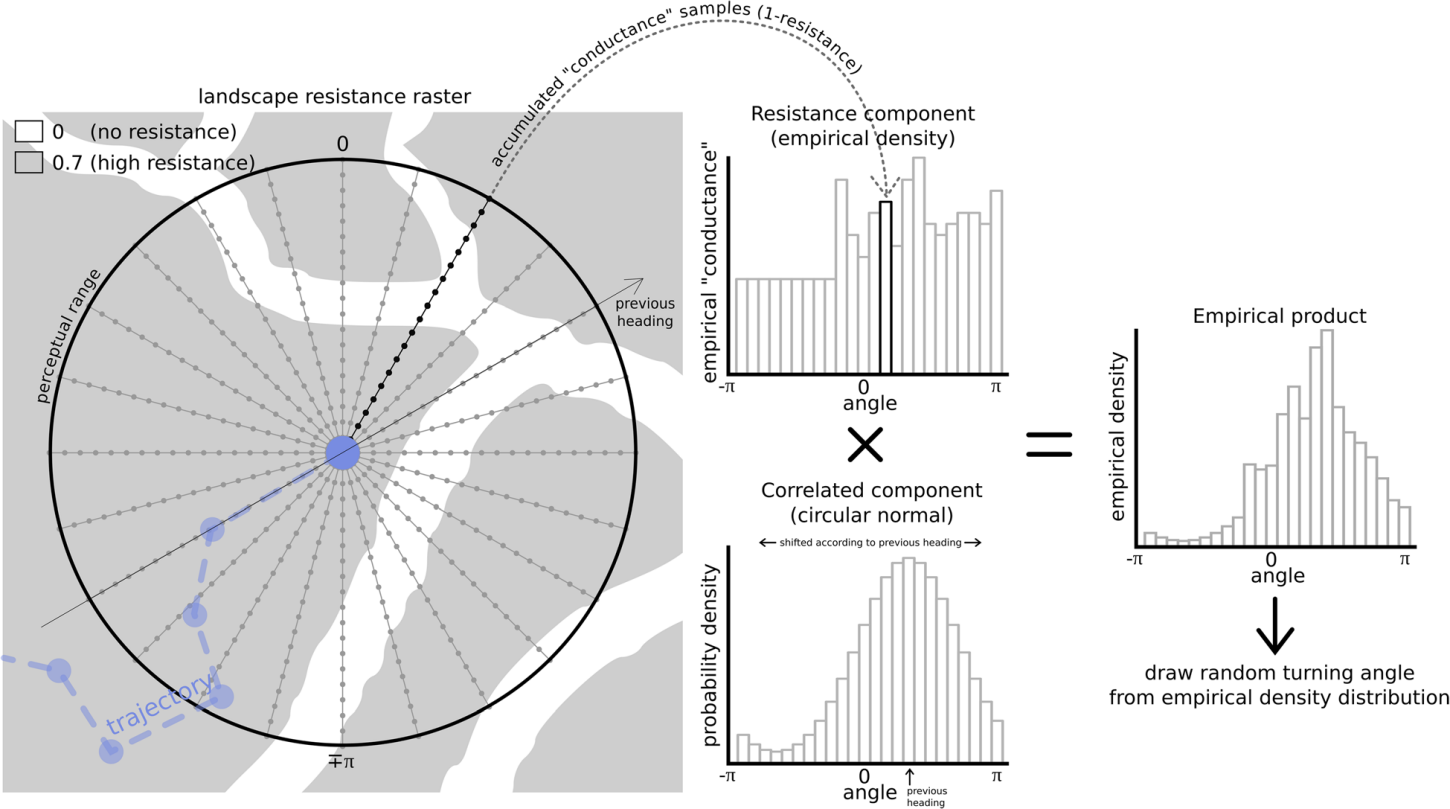
Schematic representation of SiMRiv’s algorithm for incorporating local landscape influence on movement behavior. The figure shows how resistance values of the landscape within the perceptual range are locally “perceived” by the organism whose movements are to be simulated, and how they affect its movement in respect to its decision about the heading to be taken at the next step. Note that in the current software version the landscape also influences the length of the step, which is not illustrated here (but see Additional file 1)

The current version of the algorithm rests on two basic important assumptions: a) the environment around the organism’s location influences the organism’s decision on the heading to take in the next step; and b) the environment crossed within each step influences the organism’s speed. Landscape’s physical resistance and habitat suitability are thus presently combined [31] [Additional file 1 exemplifies possible future improvements]. The simulation algorithm proceeds as follows:

1. Draw new state according to current state and the state transition matrix (e.g. [39], Table 1 - in the current version the state switching probabilities are fixed, but could be made habitat-dependent or time-dependent in a future version; for further details on potential future improvements see Additional file 1);
2. Sample resistance raster values (which are in the range [0, 1]) in samples regularly spaced along radial lines centered in the current location spanning up to the perceptual range (i.e. the radius up to which the animal perceives its surroundings [40] (see Additional file 2)) size range (Fig. 1);
3. Sum conductance values (1-resistance) of all samples in each radial line, and build an empirical distribution as a function of the angle of the respective radial line (Fig. 1);
4. Build a discrete circular wrapped normal distribution according to current state’s concentration parameter (in the range [0, 1]) and centered in the previous step heading (Fig. 1);
5. Multiply both distributions to yield a combined distribution (Fig. 1);
6. Draw a random angle using the combined distribution as the probability density;
7. Compute the actual length of the step that will be taken, as a fraction of current state’s user-defined step length. This fraction is computed by averaging the conductance of the starting and ending points in the chosen direction such that if mean resistance is 0, the actual length equals the user-defined step length, if 1, the actual length is zero;
8. Move to new location defined by the drawn angle and the actual length of step.

**Table 1:** Description of SiMRiv’s functions, excluding auxiliary functions

Function	Description
*adjustModel*	Finds approximations of the simulation input parameters able to generate simulations maximally similar to a given (real) trajectory, using the multi-objective genetic algorithm NSGA-II [46]
*generationPlot*	Plots the evolution of the optimized solutions (sets of input parameters) along the generations of the optimization algorithm during input parameter approximation to real data.
*perceptualRange*	Defines the perceptual range to be used in a behavioral state.
*sampleMovement*	Resamples a simulated movement to a lower temporal resolution and computes step-wise statistics of turning angle, step length and accumulated resistance.
*simulate*	Performs fast and spatially-explicit simulation of multistate random movements of individuals in an optional landscape resistance raster.
*species*	Creates a species to be simulated, characterized by one or more behavioral states and the respective transition matrix.
*speciesModel*	Defines a species model for which to adjust parameters based on a real trajectory, during the optimization performed by ‘*adjustModel*’.
*state, state.RW, state.CRW, state.Resting*	Defines a behavioral state to be used when defining species.
*resistanceFromShape*	Creates a resistance raster to be used in simulations, by rasterizing and combining different shapefiles.
*transitionMatrix*	Defines the state transition matrix, i.e. the probability of the individual switching from each behavioral state to another in each step.

To allow trajectories of simulated animals to properly respond to the environment, including to be constrained to linear landscapes or influenced by landscape heterogeneity, SiMRiv was designed so that movements have to be simulated with a very high temporal (and therefore spatial) resolution - possibly higher than that of many real field datasets. In this way, the behavior of the simulated animal (i.e. its realized turning angles and step lengths) is adjusted based on its context at every location in a quasi-continuous manner (Fig. 1), i.e. it makes context-based local decisions of which direction to follow at each step. SiMRiv simulations may therefore be described as multistate, locally-biased (correlated) random walks.

For the simulation algorithm to work properly, the simulated steps should be small in relation to landscape features, as the more frequently the behavior is updated, the better the landscape bias is incorporated in the movements. In particular, care has to be taken to ensure that the simulation realized step lengths are shorter than the smallest/thinnest landscape features, as this would result in the animal “skipping” portions of the landscape. To better control this, we simplified the step length input parameter to a fixed number (for each movement state) e.g. [41], with which the user defines the maximum allowable step length, instead of regarding it as a distribution from which random values are drawn in each step e.g. [39]. As a rule of thumb, we recommend the maximum step length to be set at most half the size of the smallest/thinnest landscape features. The realized step length distribution is therefore truncated in its upper limit, but this effect is minimized after downsampling simulations to a coarser frequency, similar to that of most real data see [42]. The effects of this simplification are outweighed by the flexibility of combining multiple basic movement types (Random Walk, Correlated Random Walk or Resting), thus generating compound movement types of any complexity (see below and Fig. 1 and Additional file 3: Figure C.2).

The locally-biased and high-frequency approach here presented should confer realism and high flexibility to the behavior of simulated organisms. Besides, it avoids unrealistic assumptions, such as animal omniscience and planned final destination, generally found in the least cost path (LCP) modelling approach (see also [8, 22]). These and other benefits are further illustrated in the Additional files 3 and 4, and [38]. Table 1 and Additional file 4 synthetize SiMRiv’s main functions and differences with other similar packages, respectively. Additional file 5 provides a detailed description of SiMRiv basic simulation workflow.

### Parameterizing the simulation

To simulate, users might set the input movement parameters according to available literature or expert-based criteria, or estimate them from real (telemetry) data. As SiMRiv simulates at a higher time frequency than most of real data, the statistical methods conventionally used for estimating input parameters, either bayesian e.g. [20, 39, 43] or maximum-likelihood-based e.g. [16, 44], are rather challenging to apply [45]. We are currently working on the development of a maximum-likelihood estimation method for parameterizing the simulations from real data. For now, we include a provisional function (*adjustModel*, Table 1) to approximate input parameters from real data through a pattern-oriented approach (see below). This function is built upon a consolidated heuristic numerical optimization algorithm, the genetic algorithm NSGA-II [46], which we programmed to solve the particular problem of finding simulation input parameters for simulations conducted at a possibly much higher frequency than the provided real trajectory. This function allows SiMRiv users to parameterize the high-frequency simulation model from real, low-frequency movement (telemetry) data, following a Pattern-Oriented modelling (POM) approach [47], aimed at maximizing the closeness of models to real data [36] – i.e., here, to uncover what kind of real complex high-frequency movement could have produced the observed low-frequency sample. As of current version, the function uses by default the differences in turning angle and step length distributions as metrics to quantify how similar are the simulated and observed tracks, such that during parameterization, the algorithm minimizes these metrics. However, there is room for improvement in this respect, as these two metrics lose many details that are important to characterize a trajectory. In Additional file 3 we provide a detailed explanation of this method, including a preliminary evaluation of its performance, using examples with both simulated (known) and real data, and discuss advantages and limitations of it, and how we plan to improve it.

## Results

### Examples of simulations

**Fig. 2:**
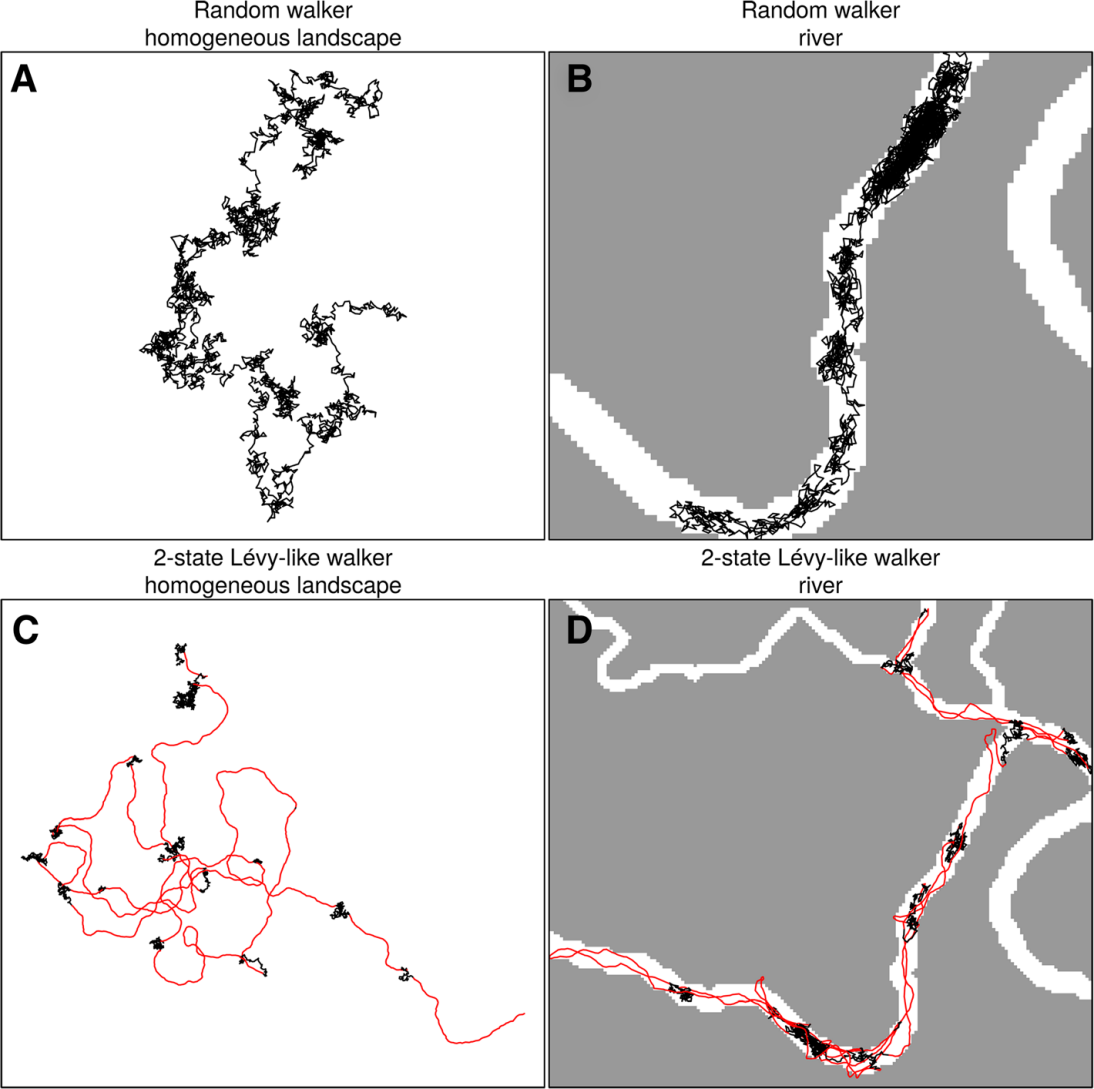
Movements simulated with SiMRiv in homogeneous lanscape and constrained to a river. Simulated movements (3000 steps) of a random walker in A) a homogeneous landscape, and B) a river; and a “Lévy-like walker”, defined a as a two-state walker with a Random Walk state (black) and a Correlated Random Walk state (red) with a high correlation and low state switching probabilities, in C) a homogeneous landscape and D) a river. Input parameters were: step length = 10, perceptual range = 200, CRW turning angle concentration = 0.95, state switching probabilities = 0.01 in both ways

**Fig. 3:**
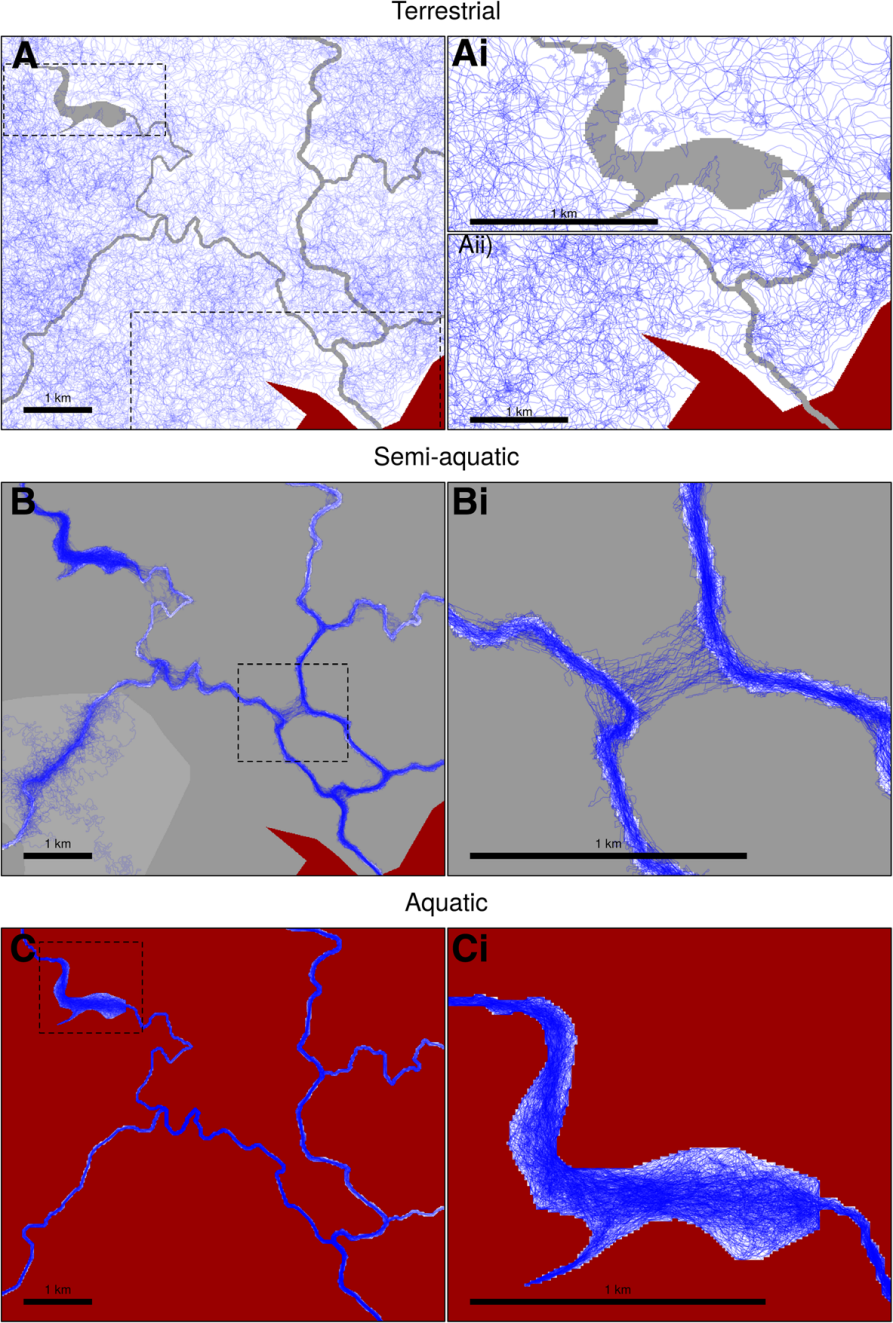
Effects of landscape. Simulated movements for three theoretical species with two-state movements (Random Walk and Correlated Random Walk) and distinct landscape dependency: **a**) a terrestrial species, completely avoiding urban areas and partially avoiding water bodies (e.g. wolves); **b**) a semiaquatic species, mostly moving along water bodies and rarely overland (e.g. amphibians, otters); **c**) an aquatic species, moving exclusively in water (e.g. fish). Landscape is shaded from white (no resistance) to dark grey (high resistance), with red corresponding to maximum resistance (i.e. where the animal cannot go). Resistance values were: a) terrestrial: water = 0.9, urban = 1, other = 0; b) semiaquatic: water = 0, forest = 0.8, urban = 1, matrix = 0.95; c) aquatic: river and dam (“water”) = 0, other = 1. Zooms on interesting resulting movement patterns are detailed on the right column. Input parameters were: step length = 10, CRW turning angle concentration = 0.95, state switching probabilities = 0.01 (RW - > CRW) and 0.002 (CRW - > RW), perceptual range = 100 (RW) and 500 (CRW)

Originally conceived to allow simulating in river and other DENs, SiMRiv’s simulation algorithm was designed to be highly flexible, being applicable to any organism (aquatic, semiaquatic or terrestrial), regardless of its space use patterns (linear, omnidirectional or their combination). Here, we demonstrate SiMRiv’s capabilities to simulate increasingly complex movements and varying input parameters. First, we show two types of movement, both simulated in a homogeneous and a linear (e.g. river) space (Fig. 2), illustrating SiMRiv’s ability to provide biologically-plausible null movement models for species occurring in homogeneous habitats and in river networks. Second, we show the effects of varying landscape resistance values on resulting simulated movements (Fig. 3). For this, we simulated two-state movements of three theoretical species along a gradient of water-dependence (terrestrial, semiaquatic and aquatic). The three species were defined by the same set of input parameters, except the resistance values assigned to the different habitat features of the landscape in which they move. SiMRiv was able to generate movements akin of the classical Lévy walk e.g. [48, 49] of a species moving nearly homogeneously in a bi-dimensional space (Fig. 3a), as well as movements with the same Lévy walk properties but strongly influenced by landscape resistance, in this case partially (Fig. 3b) and totally (Fig. 3c) constrained to river networks. Third, we show the effects of different perceptual range values. For this, we simulated movements of a theoretical semiaquatic species with 500, 2000, and 5000 m of perceptual range. Increasing perceptual range resulted in a decrease of species confinement to the water network and an increase in out-of-network movements, mostly to shortcut river meanders or to cross from one habitat patch to another (Additional file 2). Also, simulated animals with larger perceptual ranges were more attracted towards larger habitat patches and spent more time there (Additional file 2.A, Additional file 2.B). Conversely, there was an increasing tendency for small scale features to attract/repel the animal in detriment of large features, as perceptual range decreased (Additional file 2.C). Thus, under our model, although not explicitly included in simulations, patch size has an influence on both animal affinity/repulsion towards a patch, as well as the time the animal spends in it [50]. Such influence of perceptual range on species movements may have important implications in the estimation of habitat connectivity cf. [8]. However, and although further testing is needed, based on our preliminary trials it seems that the perceptual range only affects the generated movements when varied of several orders of magnitude, as varying it of only from e.g. 100 to 200 m yields very similar results (not shown). Additional file 3 provides further examples of movements generated by SiMRiv.

### Examples of SiMRiv’s possible uses

One of main SiMRiv’s potential uses is the generation of increasingly complex movements incorporating landscape effects, which could then be used as null models to test explicit Movement Ecology hypotheses [33] under a process-based, mechanistic null model framework. For instance, to assess the potential effects of landscape features (e.g. resources, roads, dams) (or their implementation/removal) on animal movement e.g. [51], researchers could use SiMRiv to simulate movements with and without the putative influence of the landscape feature/s of interest, by defining different values of affinity/repulsion (or indifference) for the landscape feature/s, and then compare the simulated movements with the observed movements. Such comparisons could be achieved using any metric quantifying the differences between the distributions of movement parameters (e.g. the Wasserstein distance between the simulated and observed step length and turning angle distributions) or directly comparing the spatial overlap of real and simulated trajectories (e.g. through kernel density estimates). Similarly, researchers could use SiMRiv to generate movements to be used as null models to test site fidelity. Finally, despite the individual-based origin, SiMRiv can also be used for analyses at population level, e.g. to assess connectivity - in [38] we provide an illustrative example using SiMRiv to predict road kill risk, discussing SiMRiv’s potentialities for connectivity research] - and, after future improvements (see Additional file 1), test for interactions. Additional file 6 provides further examples of potential uses of the software.

## Conclusions

SiMRiv fills the highlighted software gap by allowing simulation of movements in linear and heterogeneous landscapes. Importantly, SiMRiv simulates movements by accounting for local conditions - i.e. the behavior of the moving object is re-evaluated at each step, accounting for landscape effects within the perceptual range - rather than global ‘optima’ used in other approaches, such as least cost-path based analyses see [38]. The software is flexible enough to be used in linear, heterogeneous, as well as homogeneous landscapes. SiMRiv should thus significantly contribute to the study of animal movement, allowing its users to study the movement of different organisms in a variety of landscapes using the same software, algorithm and approach. Finally, SiMRiv’s simulation times are rather fast and the simulation workflow simple to understand and intuitive to use, facilitating its use among biologists. SiMRiv thus constitutes an important tool complementing existing approaches and providing a different way of addressing movement ecology and landscape ecology questions (see Additional file 4: Figure A9).

## Availability and requirements

The package is freely available under Open Source GNU GPL 3 license on CRAN, and its development version and code on Github. To install SiMRiv, select the repositories CRAN and then type: install.packages (“SiMRiv”). We welcome feedbacks, bug reports, and collaborations. Further information on SiMRiv’s functions and potential use are reported in the package’s manual and vignette.

**Project name:** SiMRiv.

**Project home page:** https://github.com/miguel-porto/SiMRiv

**Operating system(s):** Platform independent.

**Programming language:** R; C.

**Other requirements:** R 1.8.0 or higher; raster (R package).

**License:** e.g. GNU GPL v3.0.

**Any restrictions to use by non-academics:** none.

## Additional files

Additional file 1: Future software improvements. (PDF 110 kb)

Additional file 2: Effects of perceptual range on simulations. Simulated movements of a semiaquatic theoretical species (e.g. otter, moving mostly along water bodies and overland at times) with a two state movement (Random Walk and Correlated Random Walk) and varying perceptual ranges. Landscape is shaded from white (no resistance) to dark grey (very high resistance), with red corresponding to maximum resistance (i.e. where the animal cannot go). Zooms on interesting resulting movement patterns are detailed on the right column. Input parameters were: step length = 10, CRW turning angle concentration = 0.95, state switching probabilities = 0.01 (RW - > CRW) and 0.002 (CRW - > RW), perception window = 5000, 2000 and 500 m (both states). (TIF 4062 kb)

Additional file 3: Performance assessment of SiMRiv’s optimization approach. (PDF 935 kb)

Additional file 4: Comparison with other packages. (PDF 282 kb)

Additional file 5: Basic simulation workflow. (PDF 155 kb)

Additional file 6: Overview of potential uses of the software. (PDF 118 kb)

Additional file 7: R code for conducting the analyses and producing Figs. 2-3 of main text and Additional file 2. The R objects required for running these analyses and producing the figures are provided as a separate file: Additional file 7_resistance-rasters.Rdata.(Rdata + R 273 kb). (ZIP 277 kb)

Additional file 8: R code for conducting the analyses of Additional file 3. The R objects required for running these analyses are provided as a separate file: Additional file 8_otter-realdata.Rdata. (Rdata + R 11 kb). (ZIP 10 kb)

Additional file 9: Supplementary references. (PDF 85 kb)

## Abbreviations

CRW: Correlated Random Walk
DENs: Dendritic Ecological Networks
POM: Pattern-Oriented Modeling
RW: Random Walk
SiMRiv: Simulating Movements in Rivers and heterogeneous landscapes
SSFs: Step Selection Functions
