# Supplementary material for: SiMRiv: an R package for mechanistic simulation of individual, spatially-explicit multistate movements in rivers, heterogeneous and homogeneous spaces incorporating landscape bias
Source: Mov Ecol. 2019 Apr 2;7:11. doi: 10.1186/s40462-019-0154-8 (PMC6444552; doi:10.1186/s40462-019-0154-8)
Supplement: Supplementary file 1 — Future software improvements. (PDF 110 kb) [file 40462_2019_154_MOESM1_ESM.pdf]

Additional file 1: Future software improvements.

In the current software version (1.0.3), movement parameters are approximated by an experimental feature, the heuristic approximation method, which, despite appearing rather promising, is not comparable to more statistical methods traditionally used for parameter estimation, such as Maximum Likelihood estimation [Additional file 3]. For this reason, we are presently working to incorporate in SiMRiv also a more traditional Maximum likelihood parameter estimation, welcoming any help by programmers in this sense, as this task is not very straightforward [see Additional file 3 and 45 for a good overview of the challenges associated to parameter estimation in models complex such as ours]. Also, landscape's physical resistance and habitat suitability are currently represented by a single raster [as in, e.g., 31]. Instead, treating them separately, so that different movement properties could vary depending on physical resistance and habitat suitability, would be more realistic. For example, physical resistance should influence the organism's speed (i.e. step length), while habitat suitability the attraction/repulsion of the latter to specific features, by biasing its headings. In this way, an animal could be allowed to head towards a highly suitable habitat patch (e.g. a high resource density area) even if it has to cross high resistance areas, resulting in more biologically sound simulations in various situations. Currently, instead, the turning angles depend on resistance values, assuming that the individuals will avoid heading towards high resistance areas. Further, next software extension should allow the user to estimate the influence of landscape bias on local animal behavior, thus inferring habitat bias, without recurring to other packages [see Additional file 3]. Presently, this can only be set by specifying raster resistance values by expert opinion, or based on literature, or inferred using SSFs or other methods (see main text). SiMRiv's outputs would further benefit from physical resistance and habitat suitability allowed to be dynamic (i.e. to change over time), thus allowing the user to test dynamic effects on animal movement, as well as from expanding current possibilities allowing users to incorporate directional or cyclic factors, and inter-individual interactions.

Another relevant feature to include is a more formal treatment of the spatial bias. Currently, spatial bias is approximated by the concept of resistance: by specifying the landscape resistance users may obtain the effect of local spatial bias within the species perceptual range. In a next software version, users could provide spatial features (points, lines, polygons) attracting/repelling animals. This will allow users to generate standard biased random/correlated random walks [20, 29], in turn extending the types of analyses that can be performed using SiMRiv (e.g. testing infrastructures' effects or interactions, and others [cf. Additional file 6]).

Next versions could also allow the state switching to depend on habitat [e.g. 16, 39] and on time since the most recent transition (resulting in a semi-Markov process [e.g. 42-43]), account for measurement error, individual variability (i.e. including random effects), and irregular sampling [79], and provide alternative methods for parameters approximation [cf. 45 and Additional file 3]. Also, while landscape resistance is currently a global input of the simulation, future versions will allow the user to simultaneously simulate *species* with different perceptions of the same landscape. Further, future versions of SiMRiv would benefit from including the possibility to model the water flow, to account for such effect on aquatic animals' movements. A final technical, yet relevant, improvement would be to parallelize the algorithm code internally whenever possible, which would lead to a significant performance boost when conducting repeated independent simulations.

We welcome contributions and suggestions to improve the software. To facilitate this, we conceived and programmed the algorithm to allow relatively straightforward implementations of the above-mentioned and other possible extensions.
