# Supplementary figures and images for: SiMRiv: an R package for mechanistic simulation of individual, spatially-explicit multistate movements in rivers, heterogeneous and homogeneous spaces incorporating landscape bias
Source: Mov Ecol. 2019 Apr 2;7:11. doi: 10.1186/s40462-019-0154-8 (PMC6444552; doi:10.1186/s40462-019-0154-8)

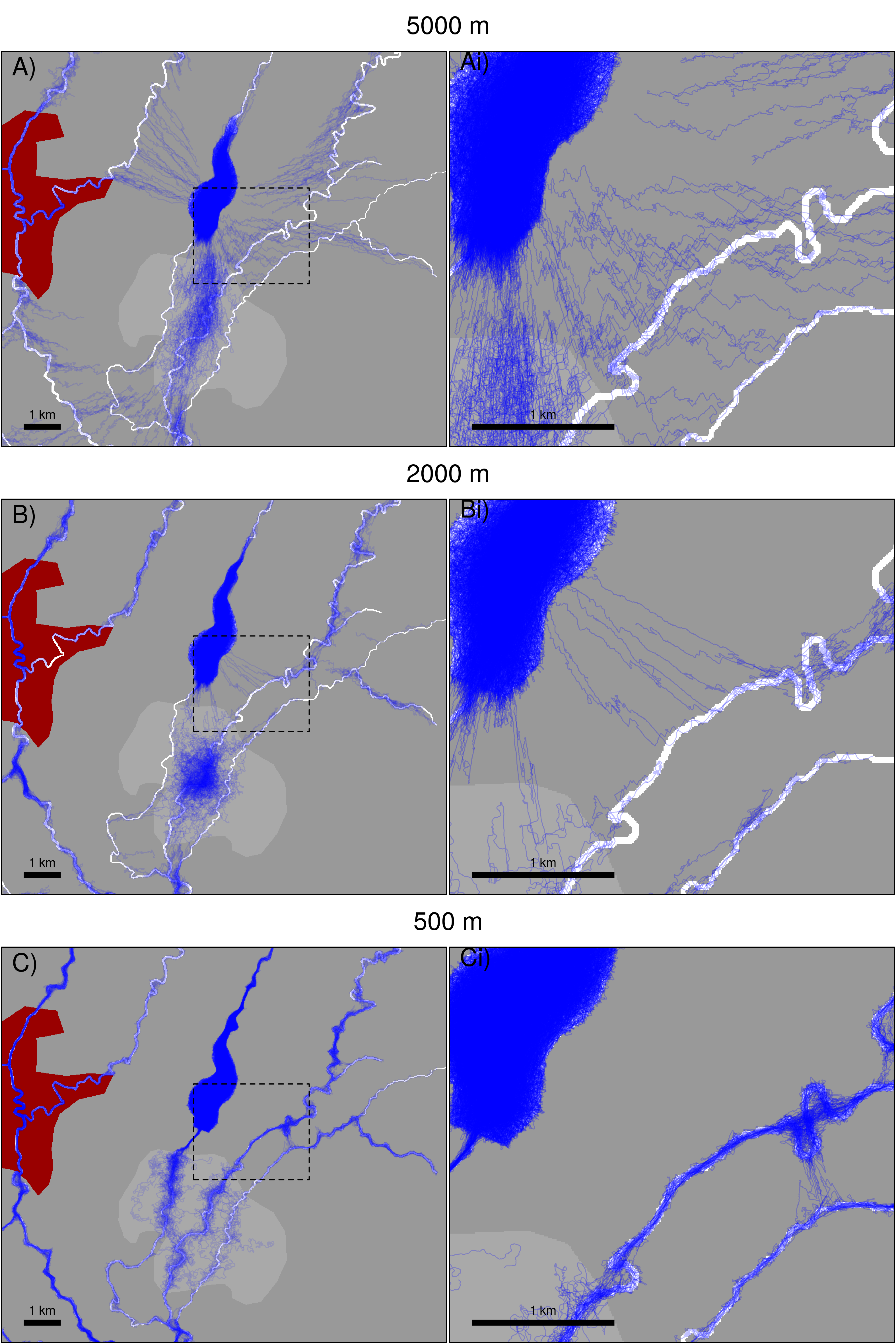

Supplement: Supplementary file 2 — Effects of perceptual range on simulations. Simulated movements of a semiaquatic theoretical species (e.g. otter, moving mostly along water bodies and overland at times) with a two state movement (Random Walk and Correlated Random Walk) and varying perceptual ranges. Landscape is shaded from white (no resistance) to dark grey (very high resistance), with red corresponding to maximum resistance (i.e. where the animal cannot go). Zooms on interesting resulting movement patterns are detailed on the right column. Input parameters were: step length = 10, CRW turning angle concentration = 0.95, state switching probabilities = 0.01 (RW - > CRW) and 0.002 (CRW - > RW), perception window = 5000, 2000 and 500 m (both states). (TIF 4062 kb) [file 40462_2019_154_MOESM2_ESM.tif]
