# Supplementary material for: SiMRiv: an R package for mechanistic simulation of individual, spatially-explicit multistate movements in rivers, heterogeneous and homogeneous spaces incorporating landscape bias
Source: Mov Ecol. 2019 Apr 2;7:11. doi: 10.1186/s40462-019-0154-8 (PMC6444552; doi:10.1186/s40462-019-0154-8)
Supplement: Supplementary file 3 — Performance assessment of SiMRiv’s optimization approach. (PDF 935 kb) [file 40462_2019_154_MOESM3_ESM.pdf]

Additional file 3*: Performance assessment of SiMRiv's optimization approach.*

Overview of the approach

The statistical methods conventionally used for estimating input movement parameters from real (telemetry) data are not easily applicable in SiMRiv's approach (see main text). Thus, while working on an alternative maximum-likelihood estimation approach (we welcome any help from programmers and statisticians in this regard), we provisionally included in SiMRiv an experimental method, built upon a consolidated heuristic numerical optimization algorithm. Numerical optimization algorithms have been used to solve very complex problems of any nature, including in genetics- or phylogenetics-oriented research [e.g. 57 and studies cited therein] and to parameterize movement models [26, 58]. SiMRiv provides a function (*adjustModel*, Table 1), built upon the genetic algorithm NSGA-II [46], which we programmed to solve the particular problem of finding simulation input parameters for simulations conducted at a possibly much higher frequency than the provided real trajectory. The function takes as input the real trajectory, the type of movement to "fit" (how many states and which parameters are fixed *a priori* or allowed to vary), and the desired simulation frequency (higher than the real), and then finds numerical approximations of the input parameters that best succeed in reproducing movements with similar pseudo-spatial patterns [sensu 59] of provided real tracking data, after downsampling back to the real data time scale. The optimization proceeds by minimizing the difference between pseudo-spatial properties of the real trajectory and those of downsampled simulated movements with different combinations of input parameters (i.e. maximizing the "fitness" of candidate solutions). As of current version (1.0.3), we quantified the "fitness" by computing the differences between the histograms of turning angles and step lengths for real and simulated movements (after downsampling), using fixed equal-range breaks. For each property (i.e. turning angle and step length), the real and simulated histograms are compared by summing the absolute differences between each corresponding pair of histogram bins (real and simulated). These histogram dissimilarity values (one value for turning angles and one for step lengths) are used as the two objectives to be minimized, taking advantage of the multi-objective nature of NSGA-II [46]. The function currently provides a third metric that can be used for fitness computation, based on the variation of turning angles along time [60]. To increase algorithm stability, given the highly stochastic nature of simulations, the histograms of simulated movements may be averaged across different realizations of the same candidate solution during its fitness evaluation. We remark that SiMRiv mechanistic simulation approach works by uncovering possible underlying general movement rules, rather than reconstructing the precise real trajectories as in other approaches that generate permutations of the observed trajectories [e.g. 61]. Thus, the simulated movements not

necessarily follow the real data in terms of spatial coordinates, neither they are supposed to do so [see also 59 and "Pros, cons and future improvements of the optimization approach"].

Assessing the performance of the optimization method

We assessed the performance of the optimization method in estimating simulation parameters using SiMRiv's own movements, generated at very high frequencies and then downsampled, as "real" data. In addition, we evaluated SiMRiv's ability to reproduce patterns similar to movements generated with existing software and to real animal movements from published datasets, in simulations conducted at a frequency several times higher than real data. We started by assessing whether the method is able to recover the true input parameters of SiMRiv simulations for three increasingly complex movement types: single-state, two-state and three-state all-CRW movements. Each state corresponds to a potentially distinct combination of turning angle concentration and step length, hence these movement types require estimating 2, 6 and 12 parameters, respectively (two parameters per state, plus the transition probabilities between each pair of states). For simplicity, though, we assumed symmetric transition probabilities for the three state movement, hence reducing the number of estimated parameters to 9. For each movement type, we respectively ran the *adjustModel* function during 200, 400 and 1000 algorithm generations with a population of 100 candidate solutions to be optimized (each candidate solution being a set of parameters), providing as "real" data a 500-relocation trajectory downsampled from a 50x higher frequency simulation with the parameters supplied in Fig. C.1. The (optimized) parameters of the Pareto-optimal solutions [46] were then compared with the true parameters used in the simulations (Fig. C.1-C.2).

Next, we evaluated SiMRiv's ability to reproduce movements obtained with existing software (multistate hidden Markov model and Lévy walks), generating "real" data using moveHMM [16] and adehabitatLT [12] packages. Finally, we examined SiMRiv's ability to reproduce real animal movements, namely telemetry data of a terrestrial (cow elk, *Cervus elaphus* [39]) and a semiaquatic (Eurasian otter, *Lutra lutra* – data preliminarily presented in [62]) species. For both assessments, we picked one "real" trajectory, ran *adjustModel* function to approximate input parameters for simulations conducted at a 50 times higher frequency (25 times in the otter case), and simulated movements using the approximated input parameters. During optimization, each candidate solution was simulated 6 times (as a reasonable compromise in terms of speed and effectiveness) and the averaged histograms used in the "fitness" computation. The resulting movements using approximated parameters were downsampled 1/50 (1/25 for the otter) to match the frequency of real data, and results (simulated movements and respective turning angle and step length histograms) were plotted for visual comparison, as well as the respective "generation" plots, for assessing algorithm convergence.

Results of the first assessment showed that the optimal solutions converged almost precisely to the true values in the single-state and two-state movements, despite the trajectory being downsampled 50 times (Figures C1.a-C.1.b). They tended to converge to a different set of parameters in the more complex 3-state movement, though (Fig. C.1.c) (see below). Remarkably, SiMRiv's high frequency simulations conducted with the parameters approximated using *adjustModel* converged to very similar patterns to the real elk and otter data and to the "real" data generated by other software (Figures C3-C4 and Figures C5-C6, respectively), even for contrasting movement types and when simulating at a 50 times higher frequency than real data. In particular, the simulations successfully reproduced, after downsampling, the pseudo-spatial patterns of real animal movements, including the notorious "encamped" and "exploratory" states of Elk data [39] (Fig. C.3) and three-state movements of Eurasian otters (Fig. C.4, see also [38]). In these cases, the algorithm converged to a single type of solution (Figures C3-C4), suggesting that it was able to recover a possible "high-frequency truth" hidden within a low frequency sample, i.e. it recovered the only one high-frequency movement type yielding samples similar to the observed low-frequency sample. This is especially evident for the Elk data (Fig. C.3), where simulations with optimized parameters reconstructed quite diverse high-frequency movement tracks that, after downsampling back to the real data time scale, showed properties very similar to those of the somewhat minimalist observed track. SiMRiv's simulations with optimized parameters were also able to reproduce both the multistate movements generated using moveHMM, as well as those of the Lévy walks generated using adehabitatLT (Figures C5-C6).

Results also show that the classical Lévy walk can readily be approximated at a higher frequency by a two-state RW-CRW movement model (Fig. 1 and Fig. C.6), corroborating previous research showing that Lévy walks may arise from composite CRWs [e.g. 30, 49]; and that a two-state movement generated by a hidden Markov model was also approximated by a simple three-state all-CRW model (Fig. C.5). SiMRiv's approach was thus able to reproduce the kinds of behavior mathematically modelled with other approaches [e.g. 16, 30, 44, 49, 63]. Notably, despite the high number of parameters that had to be approximated (12 for the three-state models), the optimization algorithm generally converged rapidly to stable values in about 500 generations, which took ca. 25 minutes in an Intel Core i7 CPU 920 @ 2.67 GHz with 8 cores. Naturally, the number and type of parameters to optimize affect the speed at which the algorithm converges, or whether it converges at all [see SiMRiv's manual for a reference and the R script in Additional file 9 for examples]. The defaults provided in the function *adjustModel* (and used herein) proved to be the best compromise between speed and quality according to our trials. Perhaps one of the most important parameters to set is the number of histogram bins to use during "fitness" computation. This determines how fine-detailed the fitness computation is - a higher number would potentially result in better fits, but our trials suggest that convergence may not be achieved

if the number is too high (probably due to the stronger impact of stochasticity). Based on our trials, a value of 7 seemed to be a good compromise.

Pros, cons and future improvements of the optimization approach

Overall, the proposed, provisional optimization method was fast and efficient, allowing to reproduce both real and simulated two-state movements in a variety of landscape types. The trials made using SiMRiv's own simulations showed, however, that when estimating a large number of parameters, the optimized parameters may not correspond exactly to the true parameters used in simulations (Fig. C.1.c). Convergence in parameters different from the true values, however, is not a serious issue. As mentioned, the focus in SiMRiv is in fact producing simulations that are actually capturing the features of the real trajectory that characterize the behavior of interest, so that such simulations can be used as null models to test movement ecology hypotheses. In this context, thus, the parameters themselves are not the focus, and should not be interpreted directly. SiMRiv’s users should in fact be aware that the simulated movements will not necessarily follow the real data in terms of spatial coordinates, neither they are supposed to do so, as the simulated individuals may move in any random direction – users may nevertheless specify fixed starting coordinates and fixed initial heading, and, in a next software version [see Additional file 1] a polygon to constrain simulations within it (e.g. a home range). Rather, users should focus on assessing whether simulations from the fitted model are actually capturing the pseudo-spatial patterns [59] of the real trajectory that they want to reproduce, i.e. the overall movement behavior characteristic of the species. Though totally unrelated, this is similar to the approach of fitting Generalized Additive Models [64], in which the fitted model parameters (coefficients) have no meaning per se, and shall not be used for interpretations, as is usually done in the simpler linear models. Based on our trials, possible reasons of the lack of complete convergence in optimized and true parameters in simulations are:

a) Some parameters can “compensate” for others. For example, a given step in the low resolution sample may be approximated, for the same number of substeps, either by a short step length and a high turning angle concentration (the individual moves straighter to the point), or with a longer step length and a lower turning angle concentration (the individual goes winding to the point). Since such details are largely lost in a low resolution sample (the norm in most telemetry studies – [42]), everything else being equal, both possibilities are adequate solutions to the problem, because both can reproduce the same pattern. However, while there may be several high-resolution possibilities for reproducing a single step, to reproduce a whole trajectory there is only a limited number of possibilities, because the simulation must reproduce the overall, joint pattern of all consecutive steps. For instance, a pattern of a series of consecutive steps

going relatively straight is highly unlikely to be obtained with a (high-resolution) winding movement pattern (low turning angle concentration). Note also that multiple distinct solutions for the same problem are the norm in multi-objective optimization problems [65], and constitute an advantage rather than a problem, allowing the user to evaluate which solution, among those producing nearly the same pattern, is more biologically plausible.

b) The optimization function (as of current version) minimizes the differences in the turning angle and step length distributions. These metrics do not capture all the details of the movement behavior, as they aggregate all steps in one histogram. Better convergence could potentially be achieved using metrics that take into account the temporal patterns of the movement, like the residence time in each state [60], or other properties, like tortuosity [7] (under development).

The presented findings show that the approximation function solved the practical problem of parameterizing a complex movement model - built by the composition of simple mechanistic bricks - to enable conducting simulations that replicated quite well the given real datasets. Further, it does so by parameterizing a model working at a much higher time frequency than real data. This is noteworthy, as it potentially enables researchers to uncover what kind of fine scale movements could have resulted in the observed coarse scale movement sample, given the overall observed pattern of the samples. The advantage of this approach, being numerical, is that it has no limits in terms of the complexity that can be incorporated - but users may prevent excessive complexity by, for example, pre-setting some switching probabilities to zero, based on empirical knowledge of the species - and no extra burden for the user when "fitting" a complex model (e.g. no need to provide initial parameter values or distributions, as in other modelling approaches). As such, it has great flexibility in reproducing realistic movements without extra costs.

For future software versions, the method will be extended to also allow estimating the influence of the landscape on animal local behavior, so that the user no longer will have to provide arbitrary resistance values for each habitat. Other possible improvements of the method include different formulations of the optimization objectives. In the examples provided, we used the global turning angle and step length distributions as criteria, but, as already mentioned, this could be either expanded by using other forms of summarizing the patterns of turning angles and step lengths, or totally replaced by using likelihood instead of pattern-based criteria. Examples of other possible pattern-based criteria would be metrics that indirectly measure the "residence time" in each behavioral state, i.e. the time that the animal spends continuously in each behavioral state - for instance, the variance of turning angles within given time segments of the trajectory [60] - as well

as path tortuosity and net squared displacement, among others. This has the potential to improve the quality of the optimized solutions by providing measures of properties that are completely lost when computing the aggregated histograms of turning angles and step lengths.

Another point that would improve parameter approximation is to enrich the simulation model with further possibilities. As of the current version, we incorporated the effects of static landscape heterogeneity, but it can easily be expanded to incorporate effects of any other factors, including spatial bias, dynamic landscapes, directional factors, individual interactions, cyclic factors, etc. This would confer it more flexibility to adjust to particular patterns that are common in animals (e.g. biased walks - cf. [20]) and that, if not accounted for, will hinder a proper convergence of the parameter approximation, as the underlying simulation model is not able to replicate such movements yet. As discussed above, the inclusion of such complexity would not represent a significant burden when “fitting” a model, as the user just needs to tell the function to account for that possibility.

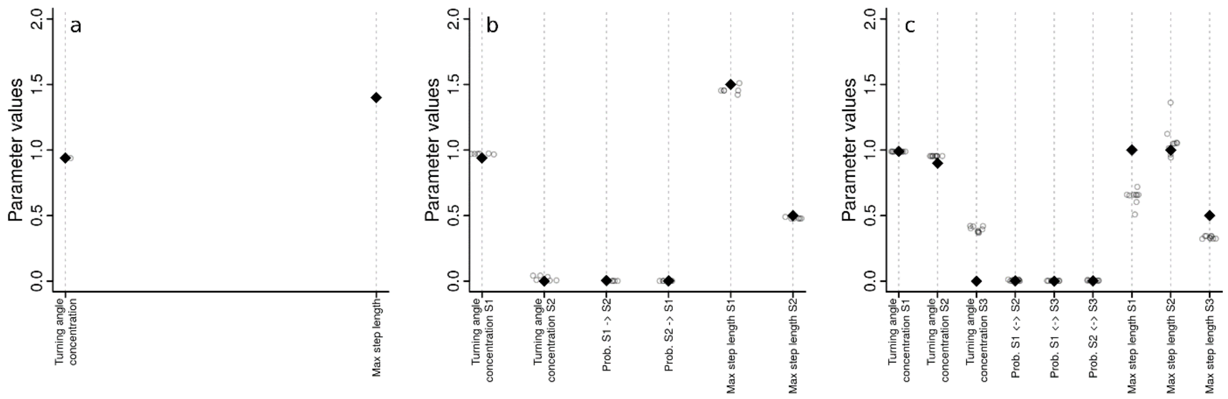

Fig. C.1. True parameters (diamonds) used for generating three simulations with SiMRiv, and respective estimated parameters (open circles) using the optimization method, for three increasingly complex movements downsampled 50 times: a) one state CRW, b) two state RW-CRW and c) three state RW-CRW-CRW. Each movement type requires estimating 2, 6 and 9 parameters (x axis) (see main text). Fig. C.2 shows the corresponding "real" and simulated movements. For the input parameters used to generate the "real" data and other minor optimization details, see the main text and the R code in Additional file 8.

Note that for the one- and two-state cases, the optimization algorithm was able to recover very well the true parameters, despite the data being downsampled 50 times, hence losing most of the detail. In the three-state case, the algorithm converged to a slightly different solution, but capable of producing the same result after downsampling See text for discussion.

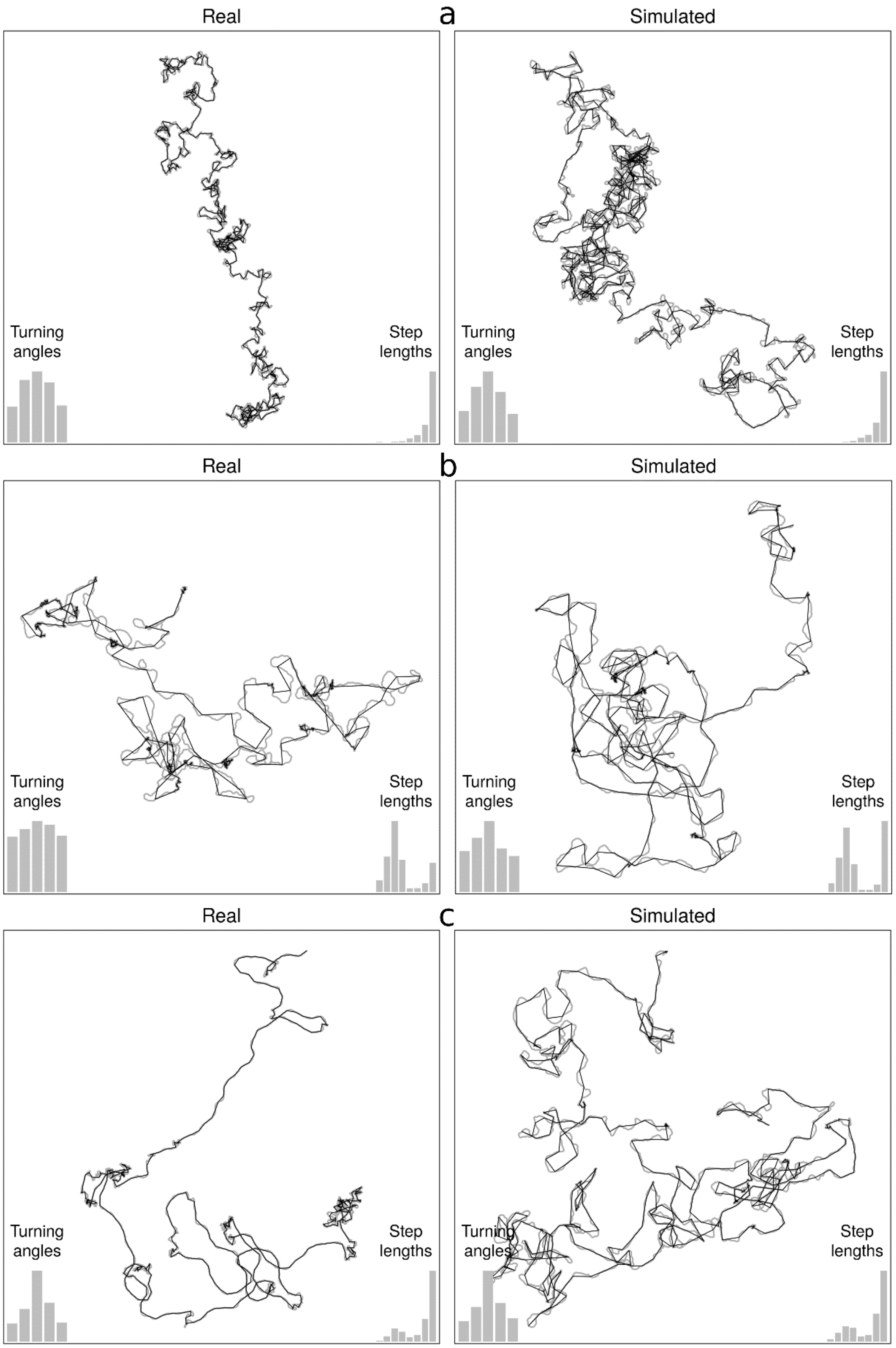

Fig. C.2. “Real” (left panels) and respective simulated movements (right panels) for three 500-step trajectories generated by SiMRiv, for which input parameters were estimated with the optimization method. The real panels depict the original movement generated by SiMRiv both at the “observed” time scale (black) and at the original simulation time scale (gray, 50 times higher frequency). Note that only the black track is known in normal circumstances. The simulated

panels depict one realization of the “fitted” movement model with the input parameters estimated from the real trajectory (black track), using the optimization algorithm, both at the simulated and observed time scales. Turning angle and step length histograms for the downsampled trajectories are shown - note that these were the criteria used during optimization, which aimed at minimizing the differences between real and simulated histograms. Note that SiMRiv's approach is to simulate movements which have the same pseudo-spatial patterns [cf. 59] than real data, irrespective of the exact spatial position, i.e. to produce different realizations of the same type of movement, and not necessarily movements that spatially match the real data (see text for details). For the input parameters used to generate the “real” data and other minor optimization details, see the text and the R code in Additional file 8.

Figures C3-C6:

In the following figures, we show: (i) the values taken by the input parameters being approximated, along algorithm’s generations, for different real and simulated datasets (real elk, real otter, simulated with moveHMM, simulated with adehabitatLT), and (ii) the real and

simulated multistate movements using the approximated input parameters for each dataset, with respective turning angle and step length histograms.

In the upper panels (i), the plots represent the 10%, 50% and 90% quantiles of the input parameters of the Pareto-optimal solutions [46] out of a population of 100 candidate solutions being optimized, along the generations of the optimization algorithm. The breadth of the 10%-90% interval indicates to what extent Pareto-optimal solutions are similar in respect to each input parameter, hence, is a measure of algorithm convergence to one type of movement. For minor optimization details, see the R code in Additional file 8.

In the lower panels (ii), the real panels depict the original movement, and the simulated panels depict three realizations (of three different solutions randomly sampled from the Pareto-optimal solution set generated by the function) of the approximated input parameters through numerical optimization. In the simulated panels, two paths are shown: the original simulated movement before frequency downsampling (in gray), and the same simulated movement after downsampling 50 times (25 times in the in the otter example), to match the frequency of real data (in black). The histograms pertain to the turning angles and step lengths after downsampling, hence they are comparable across real and simulated data. Note that these are the histograms that are used internally by the optimization algorithm to compute the “fitness” (quality) of each candidate solution, the algorithm proceeding by the minimization of the dissimilarity between real and simulated histograms (see text).

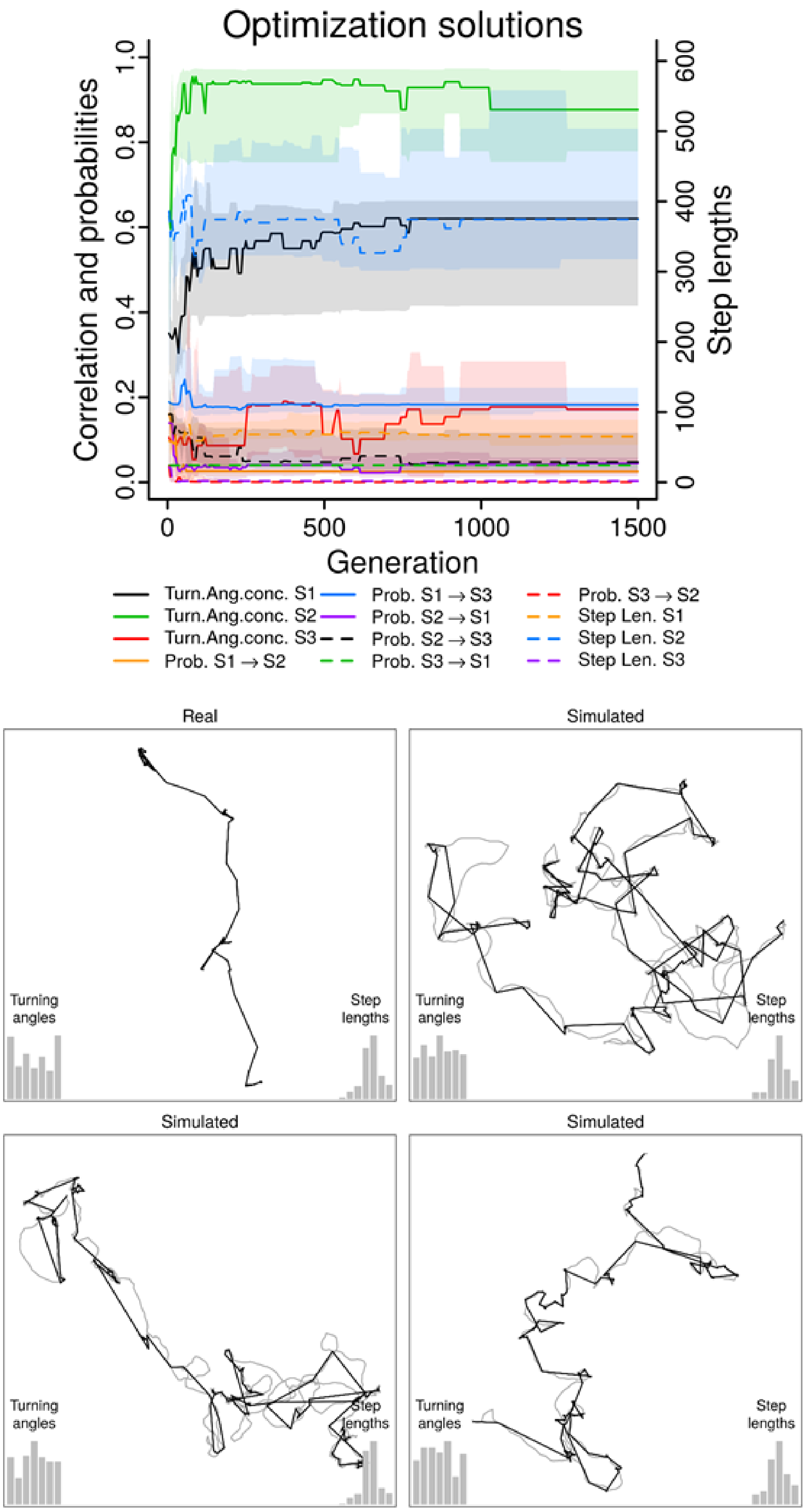

Fig. C.3. Results of optimization and respective simulations with optimized parameters for real elk data [39]. Upper panel: numerical approximation of input parameters along algorithm's generations for the Elk dataset, using as a model a flexible three-state movement model composed

by three Correlated Random Walk states (S1-S3) with variable turning angle concentrations and step lengths. Lower panels: real and simulated multistate movements for the Elk dataset, and respective turning angle and step length histograms. The real panel depicts the original movement of one elk, selected from the dataset provided in Morales et al. [39] and included in the moveHMM package [16].

Note that SiMRiv's simulations with optimized parameters were able to generate movements with very similar properties to the real Elk multistate movement, including the notorious "encamped" and "exploratory" states of Morales et al. [39].

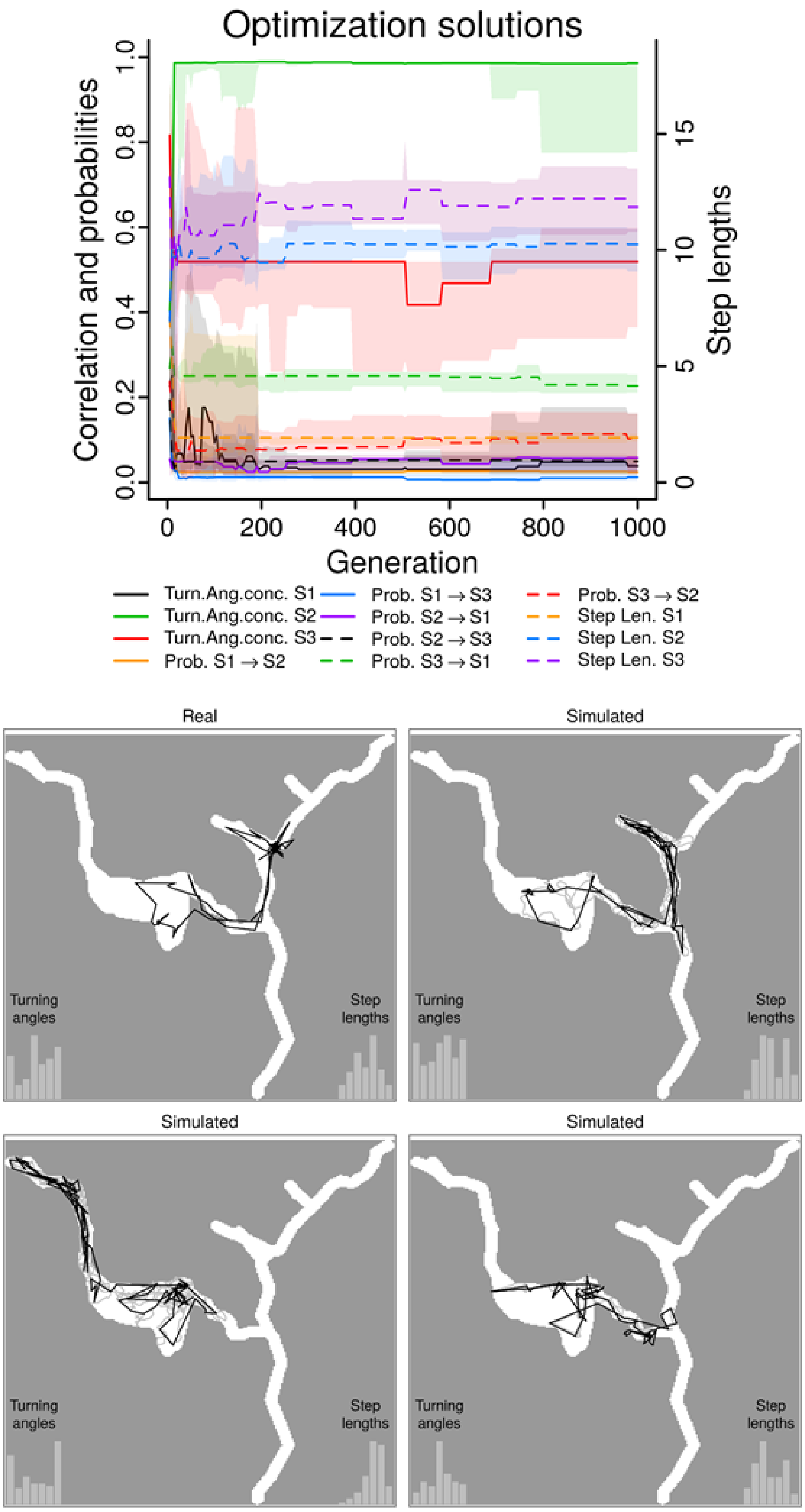

Fig. C.4. Results of optimization and respective simulations with optimized parameters for real otter data. Upper panel: numerical approximation of input parameters along algorithm's generations for an Eurasian otter (*Lutra lutra*) tagged with a GPS/GSM system [62, this study],

using as a model a flexible three-state movement model composed by three Correlated Random Walk states (S1-S3) with variable turning angle concentrations and step lengths. Lower panels: real and simulated movements for the otter dataset, with respective turning angle and step length histograms plotted over the resistance raster used during optimization. The resistance raster used to constrain movements corresponds to the real landscape where the otter was tracked and was set with the river and dam having a resistance of 0 and the matrix a resistance of 0.95.

Note that SiMRiv's simulations with optimized parameters were able to generate movements with very similar properties to the real otter movement, here approximated by a three-state CRW movement.

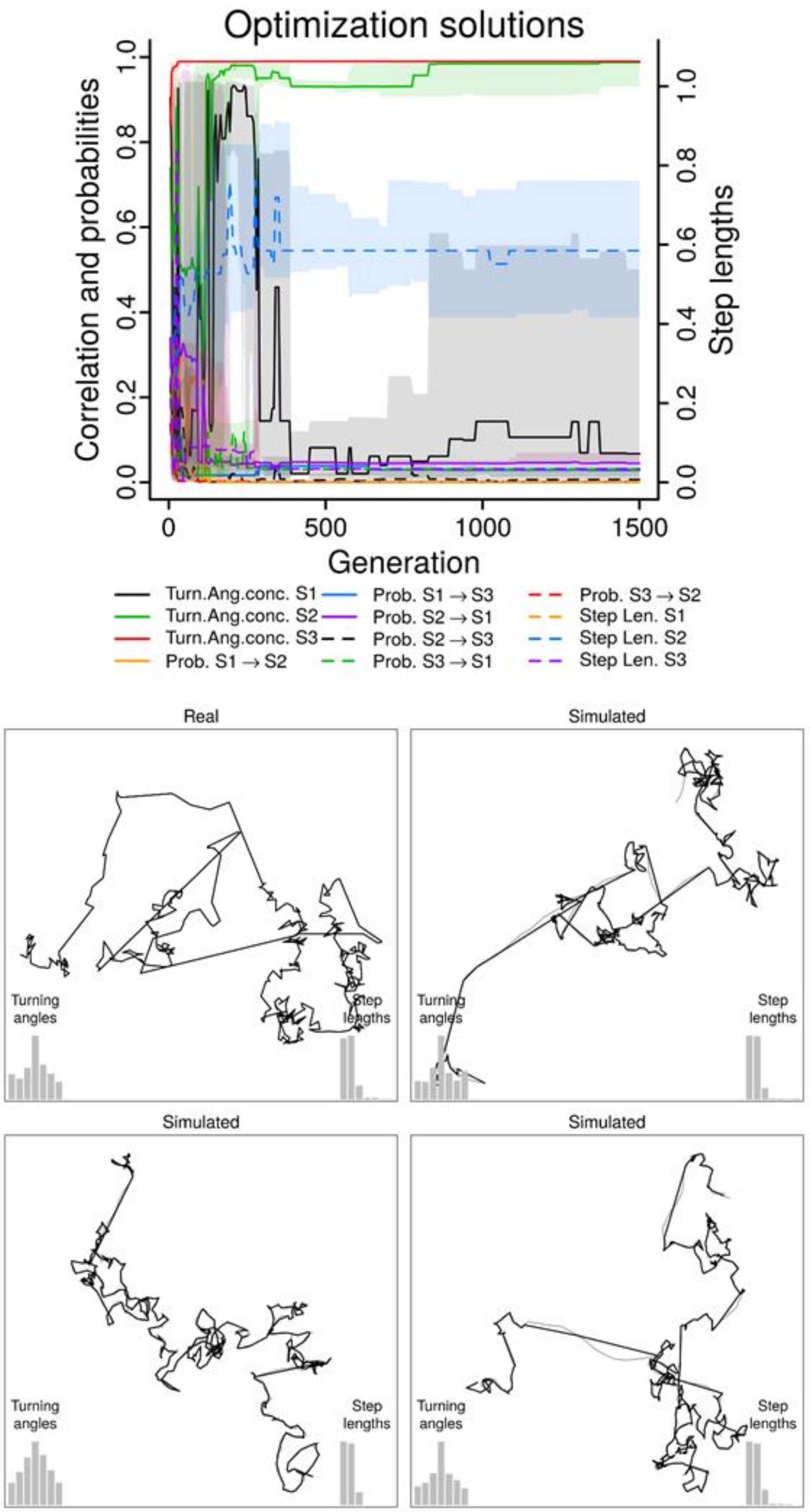

Fig. C.5. Results of optimization and respective simulations with optimized parameters for data generated with the moveHMM package [16]. Upper panel: numerical approximation of input

parameters along algorithm's generations for a 500-step two-state movement generated with the moveHMM package, using as a model a flexible three-state movement model composed by three Correlated Random Walk states (S1-S3) with variable turning angle concentrations and step lengths. Lower panels: real and simulated movements for the moveHMM-generated dataset, and respective turning angle and step length histograms.

Note that SiMRiv's simulations with optimized parameters were able to accurately reconstruct the two-state movements generated with the moveHMM package.

For the input parameters used to generate the "real" data in the moveHMM package and other minor optimization details, see the R code in Additional file 8.

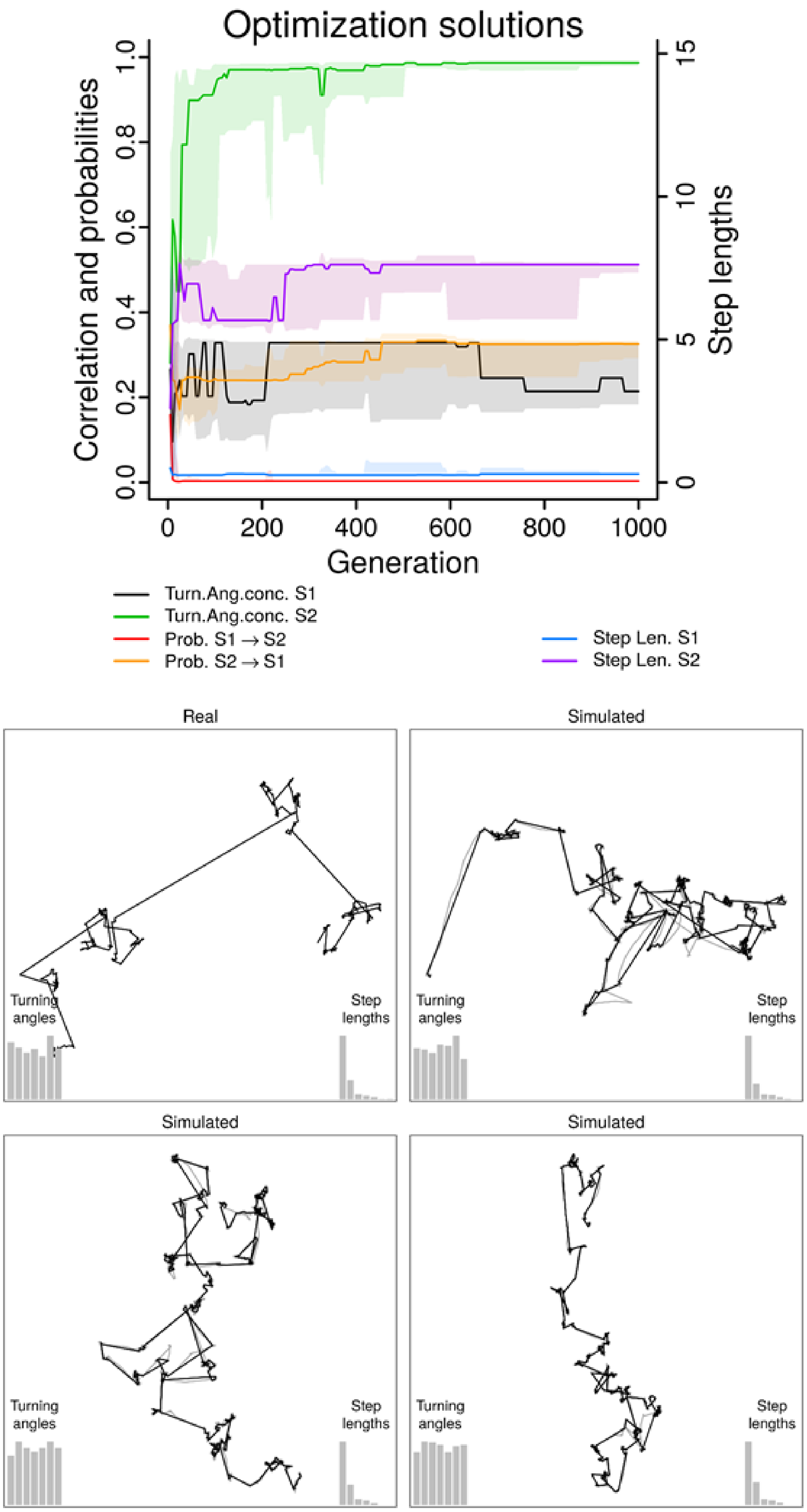

Fig. C.6. Results of optimization and respective simulations with optimized parameters for Lévy walk data generated with the adehabitatLT package [12]. Upper panel: numerical approximation

of input parameters along algorithm's generations for a 500 step Lévy walk movement generated with the *simm.levy* function of adehabitatLT package, using as a model a flexible two-state movement model composed by two Correlated Random Walk states (S1-S2) with variable turning angle concentrations and step lengths.

Lower panels: real and simulated movements for the Lévy walk movement, and respective turning angle and step length histograms.

Note that SiMRiv's simulations with optimized parameters were able to accurately reconstruct Lévy walks generated with the *simm.levy* function of adehabitatLT package.
