# Supplementary material for: SiMRiv: an R package for mechanistic simulation of individual, spatially-explicit multistate movements in rivers, heterogeneous and homogeneous spaces incorporating landscape bias
Source: Mov Ecol. 2019 Apr 2;7:11. doi: 10.1186/s40462-019-0154-8 (PMC6444552; doi:10.1186/s40462-019-0154-8)
Supplement: Supplementary file 4 — Comparison with other packages. (PDF 282 kb) [file 40462_2019_154_MOESM4_ESM.pdf]

Additional file 4: Comparison with other packages.

Other R packages allowing spatially-explicit simulation and analysis of animal movements include adehabitatLT [12], crawl [15], bsam [90], ctmm [42], moveHMM [16], and, very recently, momentuHMM [91]. However, only moveHMM, momentuHMM and SiMRiv allow incorporating behavioral state-switching mechanisms and landscape influence. SiMRiv has indeed much in common with moveHMM and momentuHMM, although there are substantial differences in the approach. First, moveHMM's and momentuHMM's main purpose is to infer the effects of multiple covariates (environmental and others) on movement by fitting a (hidden Markov) model to observed data and estimating covariate coefficients, which can later be used for simulation with the same package. SiMRiv's main purpose, on the other hand, is to provide an intuitive to use, yet very flexible, simulation tool that account for landscape constraints/influence explicitly and a priori (i.e. as an input parameter). SiMRiv generated movements can then be used as null models to test explicit Movement Ecology hypotheses [33] under a process-based, mechanistic null model framework. Second, as far as we know, SiMRiv is the only software currently available allowing to simulate spatially-explicit individual-based trajectories in linear environments or dendritic ecological networks, incorporating local effects on animal movement behavior. Third, SiMRiv moves forward in relation to the other switching movement simulation and modelling packages by providing a numerical optimization function to "fit" a complex multistate movement model to a real dataset at a much higher time frequency than that of the real data. This allows reconstructing high frequency movements that could produce patterns similar to the observed low frequency sample [92], approximating SiMRiv simulated movements with the more realistic continuous-time movement models [e.g. 42-43, 73], but without the need to delve into the complexity of the latter [38, main text, Additional files 3 and 4]. Instead, previous tools focused on modelling the sample itself. SiMRiv therefore constitutes an important complement to existing approaches, providing a different way of addressing movement ecology questions (see Fig. A9), as well as some unique features that were lacking generally (i.e. the ability to simulate movements in highly contrasted landscapes), or in previous hidden Markov modelling packages (i.e. the capability to reconstruct plausible high-frequency movements from low frequency samples). Finally, SiMRiv's processing times are rather fast and the simulation workflow simple to understand, facilitating its use among biologists.

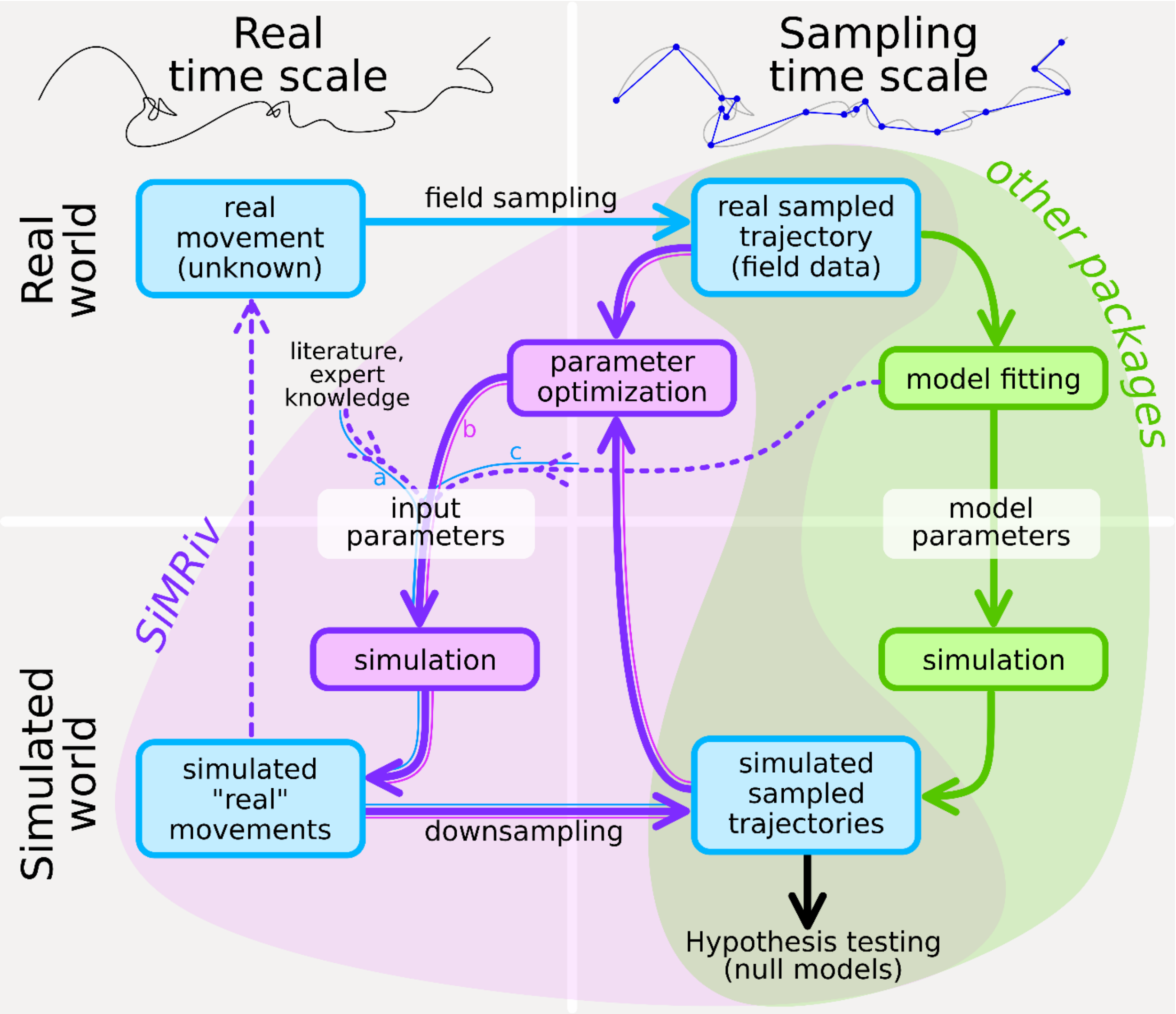

Fig. F1. SiMRiv's and other packages' (e.g. bsam, moveHMM and momentuHMM [references in main text and Additional file 9]) approaches. Solid arrows indicate data flow, dashed arrows, inference or indirect flow. Blue boxes represent data, purple and green represent SiMRiv's and other packages' algorithms respectively.

Note: SiMRiv users are not required to follow the cyclic approach of "parameter optimization" (subpath b), as input parameters may come from expert-based information or other sources (subpaths a, c).
