# Supplementary material for: SiMRiv: an R package for mechanistic simulation of individual, spatially-explicit multistate movements in rivers, heterogeneous and homogeneous spaces incorporating landscape bias
Source: Mov Ecol. 2019 Apr 2;7:11. doi: 10.1186/s40462-019-0154-8 (PMC6444552; doi:10.1186/s40462-019-0154-8)
Supplement: Supplementary file 5 — Basic simulation workflow. (PDF 155 kb) [file 40462_2019_154_MOESM5_ESM.pdf]

Additional file 5: Basic simulation workflow

Define species

The input parameters characterizing the movements (Table A.1) are abstracted by the concept of "species" (could be a specific gender, a population, or whatever the subject of the simulation is): a list of behavioral states and the switching probabilities between them (Table A.1). Each state is defined by the turning angle concentration (correlation between the headings of two consecutive steps), the maximum step length allowed, and the perceptual range (Table A.1). Users might set the input parameters according to available literature, expert-based criteria, or estimate them from real data. Currently, three elementary types of states are allowed: Random Walk [29], Correlated Random Walk [52], and resting. With these bricks, it is possible to obtain compound movement types of any complexity, similar to how Lévy walks may arise from composite CRWs [cf. 30, 49].

Building a resistance landscape (optional)

This step may be skipped for simulations in homogeneous environments (e.g. bears or fish moving at 360º in a homogeneous forest or sea, respectively). For linear or heterogeneous landscapes, however, a resistance landscape must be provided. This is a raster with pixel values ranging from 0 (no resistance) to 1 (maximum resistance), defining the permeability/affinity of the organism to each pixel (Table A.1). Currently, for technical reasons, it is not possible to estimate the values representing the influence of landscape features on species movements, but such feature is a planned future improvement of the *adjustModel* function. Presently, users have different options to set the landscape resistance values [cf. 32]: expert-based opinion, literature, or inference from real data using, for example, step selection functions [10, 34, 53] or other existing approaches [e.g. 16, 22, 54], including SiMRiv's own feature *adjustModel* (this will only be possible after next extensions of the software [see Additional file 1]). SiMRiv provides a utility function (*resistanceFromShape*) to create the resistance raster from vector data (shapefiles), allowing the user to stack multiple shapefiles (point, line or polygon) into one raster and define pixel values based on a shapefile field or constant value (Table 1).

Simulate

Simulation proceeds by providing a list defining the species for each individual to be simulated (may be only one) and the number of steps to simulate (Table 1). Optionally, the resistance raster and the initial coordinates and heading for each individual can be provided. As in other approaches [e.g. 16, 39], location error is assumed to be negligible, and simulation steps are currently assumed to be regularly spaced in time, although they may be resampled to uneven time lags afterwards. Each simulation may be conducted with any number of individuals belonging to

one or more species. Currently, the output is a matrix with coordinates and the behavioral state, for each step and each simulated individual.

Processing times are fast, as the core of the algorithm, which is computationally intensive, is implemented in C programming language, albeit being fully integrated in an R package. Typical running times for conducting 1000 simulations with 10 000 steps each, of two-state movement trajectories in a river network are of ca. 3 minutes in an Intel Core i7 CPU X990 @ 3.47 GHz. Note that the algorithm, in the current version [see Additional file 1 for future improvements], runs in a single core, but when conducting multiple independent simulations, this can easily be parallelized using the standard R package for parallel computing ('parallel'). Some caution when parallelizing simulations using large raster files by means of the 'parallel' package must be taken, however, as the computer memory may not be enough.

Analysing movements

To make high-frequency simulated movements comparable with real data (normally collected at lower frequency), a function (*sampleMovement*) is provided. The function downsamples movements and, simultaneously, computes emergent basic metrics of each downsampled step: step length, turning angle and accumulated resistance values along the resampled steps (Table 1). These metrics can then be used to compare simulated and true movements [e.g. Additional file 3 and 38].

Table A.1. Input parameters of the simulation algorithm and their description. Practical details on how to define them are provided in the package’s vignette [55].

Parameter	Scope	Optional	Range	Description
Maximum	Behavioral	yes	[0, Inf[	Defines the maximum length of the step that the

step length	state	(defaults to 1)		animal can take. The realized step length at each step is a fraction of this value, proportional to the resistance that must be crossed in the step. This value should be lower than the size/width of the smallest landscape features.
Turning angle concentration	Behavioral state	no	[0, 1]	Defines the amount of correlation between the angles of two consecutive steps. 0 means no correlation, yielding a random walk, 1 means full correlation i.e. the angle does not change between steps. Currently, the software uses a wrapped normal distribution for angles, with its standard deviation parameter being equal to $\sigma = \sqrt{-2 \times log(concentration)}$ [56]
Size of perceptual range	Behavioral state	yes	[0, Inf[	Defines the radius of the circle, centered in each location, within which the animal perceives the environment, i.e. the "circular slice" of the landscape which influences the animal's decisions at each location.
Transition matrix	Species	no	[0, 1] (each element of the matrix)	Defines the probabilities of switching between all pairs of behavioral states. State switching is evaluated at the beginning of each step.
Resistance raster	Global	yes	[0, 1] (each pixel)	Defines the "permeability/affinity" of the animals to each pixel. This is reflected both in the realized step lengths and in the choice of the heading in each step.
