# Supplementary material for: SiMRiv: an R package for mechanistic simulation of individual, spatially-explicit multistate movements in rivers, heterogeneous and homogeneous spaces incorporating landscape bias
Source: Mov Ecol. 2019 Apr 2;7:11. doi: 10.1186/s40462-019-0154-8 (PMC6444552; doi:10.1186/s40462-019-0154-8)
Supplement: Supplementary file 6 — Overview of potential uses of the software. (PDF 118 kb) [file 40462_2019_154_MOESM6_ESM.pdf]

Additional file 6: **Overview of potential uses of the software.**

We originally developed SiMRiv to help filling the highlighted gap of a tool allowing the simulation of spatially-explicit, individual movements that may be constrained to linear habitats or influenced by landscape. As such, SiMRiv should be of particular interest to ecologists working with dendritic/highly structured [e.g. 17, 22, 66-67] or heterogeneous [e.g. 25-26, 48, 5061] landscapes. Importantly, SiMRiv simulation algorithm was built to be highly flexible, being thus applicable to any organism (aquatic, semiaquatic or terrestrial), regardless of its space use patterns (linear, omnidirectional or a mixture of both). Using the same algorithm and framework, users can in fact simulate and analyse movements of organisms living in homogeneous (e.g. ungulates or fish in a homogeneous forest/sea, or a gas particle in a homogeneous space), heterogeneous (e.g. semiaquatic mammals using both river network and matrix), and even highly contrasted (e.g. fish in a river) landscapes. This flexibility will expectably facilitate the realization of comparative studies across distinct species and habitats.

Here, we list several examples of potential use of the package, from mechanistic null movement models generation, to assessing site fidelity, connectivity, and interactions. Perhaps one of the most powerful examples of use is indeed to generate increasingly complex mechanistic multistate null models incorporating landscape effects, which could then be used to test ecological hypotheses on Movement Ecology [33]. Examples include testing the influence, on animal movement, of: (i) landscape features, such as resources [18-19, 68], or roads [34, 38, 69] and other linear infrastructures [70-71] - but note that the tool can be used to test the potential effects of any landscape feature, irrespective of its geometry, size or putative (positive or negative) effect (e.g. dams [72]); (ii) species traits [73-74]; and (iii) territoriality [3, 6, 27]. Biologists and wildlife managers might, for instance, assess the potential effects of landscape features (or their implementation/removal) on species movement behavior [e.g. 51] by defining different values of affinity/repulsion (or indifference) for the landscape feature/s of interest, simulating movements with and without the putative influence of such landscape feature/s, and comparing the simulated distributions of movement parameters with the observed values (e.g. telemetry data). This is similar to what is commonly done within the Step Selection Functions (SSFs) framework [see 75 for a comparison of results obtainable using SSFs and biased CRWs]. Quaglietta et al. [38] provide an illustrative example using SiMRiv to assess wildlife-vehicle interactions and predict road kill risk based on real otter movements data.

Importantly, the incorporation of perceptual range [Additional file 2] might allow ecologists using SiMRiv to assess the distance at which animals respond to specific landscape features (e.g., roads,

lakes, city perimeters, other individuals of same/different species, resting sites, foraging sites, dumps). Further, researchers could be interested in using dynamic, instead of stationary, landscape scenarios (e.g. current and forecasted species ecological niches or land uses), for example to assess the effects of predicted climate or land use change on species movement patterns, thus evaluating their persistence and ability to disperse, migrate, and adapt to new environments [e.g. 58].

The software should also be particularly useful as a tool to assess site fidelity. In fact, we note that most of site fidelity assessments are generally performed on terrestrial species [e.g. 6, 27, 76], perhaps due to the software gap highlighted in this article, whereas researchers working with species living in river networks have long used rather simplistic methods to test for site fidelity [e.g. 28, 77]. Researchers working with aquatic and semiaquatic species occurring in lotic habitats could find in SiMRiv a tool to generate spatially-explicit movements along streams and rivers, and therefore to obtain more realistic distributions of step lengths and turning angles, facilitating rigorous assessment of site fidelity. SiMRiv could also meet the interest of researchers working with point pattern analyses on other linear (non-uniform) networks, such as roads [e.g. 78].

Further, although conceived with an individual-based approach, SiMRiv can be used to simulate several individuals over time, to investigate processes acting at population level (upscaling [79]), e.g. connectivity, species persistence and dispersal in heterogeneous landscapes. Despite the individual-based origin, thus, SiMRiv might constitute an alternative (or a complement) to other methods generally used to assess processes related with movement at population level and landscape connectivity [cf. 7], such as the least cost path (LCP) approach – with the advantage of avoiding unrealistic assumptions, such as animal omniscience and planned final destination, generally found in the LCP modelling approach [see 8, 22] - and others of this kind [e.g. 8, 22, 80-82] [e.g. 38]. Also, ecologists interested in the effects of dendritic ecological network geometry could use SiMRiv to generate movements constrained in river landscapes with varying degrees of 'branchiness', to test the intrinsic effects of river configuration on animal movements and river connectivity [e.g. 17, 67, 83]. Moreover, by allowing, in future software versions [see Additional file 1], to specify attraction/repulsion among moving individuals, SiMRiv will also allow to assess interactions [e.g. 3, 27, 84] and group movement behavior [e.g. 85].

In summary, SiMRiv should help understanding ecological processes related to movement, and, as such, be of interest to researchers using most of the current, widely used approaches for studying movement [e.g. 18, reviews in 79 and 86]. Moreover, it holds potential for applicability in fields as varied as behavior, management, movement and landscape ecology, disease and invasive species spread, conservation, and population dynamics. Eventually, it should be of

interest to scientists working in all areas requiring the simulation of individual random movements, including anthropology [87], cell biology [1], fishing [88], molecular ecology [4, 89], paleontology [5], and tumor angiogenesis [2].
