# Supplementary material for: SiMRiv: an R package for mechanistic simulation of individual, spatially-explicit multistate movements in rivers, heterogeneous and homogeneous spaces incorporating landscape bias
Source: Mov Ecol. 2019 Apr 2;7:11. doi: 10.1186/s40462-019-0154-8 (PMC6444552; doi:10.1186/s40462-019-0154-8)
Supplement: Supplementary file 9 — Supplementary references. (PDF 85 kb) [file 40462_2019_154_MOESM9_ESM.pdf]

Additional file 9: Supplementary references.

52. Kareiva PM, Shigesada N. Analyzing insect movement as a correlated random walk. Oecologia. 1983;56:234–8.

53. Potts JR et al. Predicting local and non-local effects of resources on animal space use using a mechanistic step selection model. Methods in ecology and evolution. 2014;5:253–62.

54. Signer J, Ovaskainen O. Detecting the influence of environmental covariates on animal movement: a semivariance approach. Methods in Ecology and Evolution. 2017;8:561–70.

55. Porto M, Quaglietta L. 'SiMRiv' (version 1.0.1) An R package for simulation and analysis of spatially-explicit individual multi-state (animal) movements in any landscape. https://cran.r-project.org/web/packages/SiMRiv/vignettes/SiMRiv.pdf. 2017.

56. Lund U, Agostinelli C. Circstats: circular statistics, from "topics in circular statistics"(2001). S-plus original by Lund, U. R port by Agostinelli, C. R package version 0.2-4. 2015.

57. Zambrano-Vega C, et al. MO-Phylogenetics: a phylogenetic inference software tool with multi-objective evolutionary metaheuristics. Methods in Ecology and Evolution. 2016;7:800–5.

58. Watkins KS, Rose KA. Simulating individual-based movement in dynamic environments. Ecological Modelling. 2017;356:59–72.

59. Bauduin S, et al. Overcoming challenges of sparse telemetry data to estimate caribou movement. Ecological modelling. 2016;335:24–34.

60. Calenge C. Analysis of animal movements in R: the adehabitatLT Package. In *R Package Version 0* (Vol. 3, p. 21). https://cran.r-project.org/web/packages/adehabitatLT/vignettes/adehabitatLT.pdf. 2015.

61. Proulx CL, et al. Improving the realism of random walk movement analyses through the incorporation of habitat bias. Ecological modelling. 2013;269:18–20.

62. Quaglietta L, et al. GPS telemetry & otters: preliminary experiences from three pioneer European projects. Wild Musteloid Conference, Oxford University, UK, March 2013. 2013.

63. Langrock R, King R, Matthiopoulos J, Thomas L, Fortin D, Morales JM. Flexible and practical modeling of animal telemetry data: hidden Markov models and extensions. Ecology. 2012;93:2336–42.

64. Wood SN. Stable and efficient multiple smoothing parameter estimation for generalized additive models. Journal of the American Statistical Association. 2004;99:673–86.

65. Kennedy MC, et al. Informed multi-objective decision-making in environmental management using Pareto optimality. Journal of Applied Ecology. 2008;45:181–92.

66. Peterson EE, et al. Modelling dendritic ecological networks in space: an integrated network perspective. Ecology Letters. 2013;16:707–19.

67. Fronhofer EA, Altermatt F. Classical metapopulation dynamics and eco-evolutionary feedbacks in dendritic networks. Ecography. 2017;40:1455–66.

68. Queiroz N, et al. Ocean-wide tracking of pelagic sharks reveals extent of overlap with longline fishing hotspots. Proceedings of the National Academy of Sciences. 2016;113:1582–7.

69. Roever CL, et al. Grizzly bear movements relative to roads: application of step selection functions. Ecography. 2010;33:1113–22.

70. Latham ADM, et al. Movement responses by wolves to industrial linear features and their effect on woodland caribou in northeastern Alberta. Ecological Applications. 2011;21:2854–65.

71. Borda-de-Água L, et al. Railway Ecology. – Springer. https://link.springer.com/book/10.1007/978-3-319-57496-7. 2017.

72. Noonan MJ, et al. A quantitative assessment of fish passage efficiency. Fish and Fisheries. 2012;13:450–64.

73. van de Kerk M, Onorato DP, Criffield MA, Bolker BM, Augustine BC, McKinley SA, Oli MK. Hidden semi-Markov models reveal multiphasic movement of the endangered Florida panther. Journal of Animal Ecology. 2015;84:576–85.

74. Spiegel O, et al. What's your move? Movement as a link between personality and spatial dynamics in animal populations. Ecology letters. 2017;20:3–18.

75. Duchesne T, Fortin D, Rivest L-P. Equivalence between step selection functions and biased correlated random walks for statistical inference on animal movement. PloS one. 2015;10: e0122947.

76. Mahoney PJ, Young JK. Uncovering behavioral states from animal activity and site fidelity patterns. Methods in Ecology and Evolution. 2017;8:174–83.

77. Kemp PS, et al. Challenging convention: the winter ecology of brown trout (*Salmo trutta*) in a productive and stable environment. Freshwater Biology. 2017;62:146–60.

78. Okabe A, et al. A kernel density estimation method for networks, its computational method and a GIS-based tool. International Journal of Geographical Information Science. 2009;23:7–32.

79. Hooten MB, Johnson DS, McClintock BT, Morales JM. Animal movement: statistical models for telemetry data. CRC Press; 2017.

80. Landguth EL, et al. UNICOR: a species connectivity and corridor network simulator. Ecography. 2012;35:9–14.

81. Landguth EL, et al. CDMetaPOP: an individual-based, eco-evolutionary model for spatially explicit simulation of landscape demogenetics. Methods in Ecology and Evolution. 2017;8:4–11.

82. Ribeiro JW, et al. Landscape Corridors (LSCorridors): a new software package for modeling ecological corridors based on landscape patterns and species requirements. Methods in Ecology and Evolution. 2017;8: 1425–1432.

83. Fagan WF, et al. Riverine landscapes: ecology for an alternative geometry. – In: Cantrell, S. et al. (eds.), Spatial ecology. CRC Press; 2009. p. 85–100.

84. Spiegel O, et al. Socially interacting or indifferent neighbours? Randomization of movement paths to tease apart social preference and spatial constraints. Methods in Ecology and Evolution. 2016;7:971–9.

85. Langrock R, et al. Modelling group dynamic animal movement. Methods in Ecology and Evolution. 2014;5:190–9.

86. Patterson TA, Thomas L, Wilcox C, Ovaskainen O, Matthiopoulos J. State–space models of individual animal movement. Trends in ecology & evolution. 2008;23:87–94.

87. Raichlen DA, et al. Evidence of Lévy walk foraging patterns in human hunter–gatherers. Proceedings of the National Academy of Sciences. 2014;111:728–33.

88. Gloaguen P, et al. An autoregressive model to describe fishing vessel movement and activity. Environmetrics. 2015;26:17–28.

89. Hoban S. An overview of the utility of population simulation software in molecular ecology. Molecular ecology. 2014;23:2383–401.

90. Jonsen I, et al. State-space models for bio-loggers: a methodological road map. Deep Sea Research Part II: Topical Studies in Oceanography. 2013;88:34–46.

91. McClintock BT, Michelot T. momentuHMM: R package for analysis of telemetry data using generalized multivariate hidden Markov models of animal movement. – https://128.84.21.199/pdf/1710.03786.pdf. 2017.

92. Parton A, Blackwell PG. Bayesian Inference for Multistate ‘Step and Turn’ Animal Movement in Continuous Time. Journal of Agricultural, Biological and Environmental Statistics. 2017;22:373–92.
